# Advancing *in vitro* vascular wall modelling using digital light processing to study hyperglycemia-driven cell changes

**DOI:** 10.3389/fbioe.2026.1677364

**Published:** 2026-02-04

**Authors:** Ianina Pokholenko, Marguerite Meeremans, Sandra Van Vlierberghe, Nele Pien, Catharina De Schauwer

**Affiliations:** 1 Polymer Chemistry & Biomaterials Group, Centre of Macromolecular Chemistry (CMaC), Department of Organic and Macromolecular Chemistry, Ghent University, Ghent, Belgium; 2 Department of Translational Physiology, Infectiology and Public Health, Faculty of Veterinary Medicine, Ghent University, Merelbeke, Belgium; 3 Department of Cell Regulatory Mechanisms, Institute of Molecular Biology and Genetics of NAS of Ukraine, Kyiv, Ukraine

**Keywords:** acrylate-endcapped urethane-based polyethylene glycol, digital light processing, gelatin methacryloyl, hyperglycemia, vascular wall model

## Abstract

**Background:**

Metabolic syndrome is a pathological state, frequently associated with type 2 diabetes, which is marked by abdominal obesity, impaired insulin action, hypertension, and vascular wall changes. Similar to humans, horses can suffer from equine metabolic syndrome. A representative *in vitro* vascular wall model is needed to study its pathophysiology and develop novel treatments for both human and equine patients.

**Methods:**

In this study, scaffolds manufactured via digital light processing (DLP) exploiting an acrylate-endcapped urethane-based polymer precursor with a polyethylene glycol backbone (AUP2PEG) were coated with collagen or gelatin derivatives. Their cell-interactive properties were evaluated using equine mesenchymal stromal cells (MSC) and endothelial cells (EC). Coating was performed using either UV-induced photopolymerization of gelatin methacryloyl (GelMA) on the surface of the DLP-printed scaffold or physisorption of type I atelocollagen.

**Results:**

The GelMA coating formed a thin, uniform layer on the scaffold surface and improved the cytocompatibility of DLP-printed AUP2PEG-based scaffolds for EC and MSC. Furthermore, they permitted MSC trilineage differentiation. To mimic the endothelial damage occurring in metabolic syndrome conditions, the GelMA-coated AUP2PEG scaffolds were incubated in high glucose culture conditions. Short-term cell culture in these conditions significantly reduced the number of viable EC. In contrast, the short-term culture of MSC in these conditions did not result in a similarly deleterious impact on cell viability.

**Conclusion:**

In conclusion, GelMA-coated DLP-printed AUP2PEG scaffolds facilitate the growth of EC and MSC. Furthermore, exposing EC cultured on the developed scaffolds to hyperglycemic culture conditions negatively affects the viability of EC, comparable to what is observed in two-dimensional culture conditions.

## 1 Introduction

The prevalence of metabolic syndrome (MetS), defined by WHO as a pathological condition characterized by abdominal obesity, insulin resistance, hypertension, and hyperlipidaemia, is constantly rising (Saklayen, 2018). One of its possible outcomes is type 2 diabetes, which has reached the scale of a global pandemic affecting nearly 6.5% of the global population, and the number is expected to increase to 10.4% by 2040 (Saklayen, 2018; Khan et al., 2020; Lu et al., 2024). Obesity, dyslipidaemia, and metabolic dysfunction-associated steatotic liver disease usually precede or accompany the onset of type 2 diabetes in MetS. With the hepatic insulin resistance considered as one of the main drivers contributing to impaired insulin signalling (Niranjan et al., 2023). According to literature, it mainly impacts fasting and the early phase postprandial plasma glucose levels (Yang et al., 2025). In addition to the development of insulin resistance, type 2 diabetes is also characterised by a progressive decline in the ability of pancreatic β-cells to secrete adequate levels of insulin. All these factors together result in elevated blood glucose levels or hyperglycemia (in humans as blood glucose level higher than 7 mM, and in critical cases reaching 33.3 mM or higher (Pasquel and Umpierrez, 2014; Lu et al., 2024)). Continuous hyperglycemia and atherosclerosis are considered major initiators of the development of both diabetic macro- (cardiovascular, cerebrovascular, and peripheral arterial disease), and microvascular complications (retinopathy, nephropathy, and neuropathy) (Lu et al., 2023).

Similar to humans, horses can suffer from equine metabolic syndrome (Morgan et al., 2015), which is commonly diagnosed between the age of 5 and 15 years (Ertelt et al., 2014), and is also associated with obesity, insulin dysregulation (hyperinsulinemia or abnormal glycemic and insulinemic responses to oral or intravenous glucose or insulin challenges) (Frank et al., 2010)), and vascular wall changes. In horses, mainly small caliber vessels in the hoof are affected, leading to laminitis. Although hyperinsulinemia, which is also strongly linked to type 2 diabetes in humans (Thomas et al., 2019), is one of the models used to induce laminitis under experimental conditions (Ragno et al., 2019), persistent hyperglycemia, in horses defined as blood glucose concentrations above 7.3 mM (Hollis et al., 2007), is less common. However, it has been demonstrated that continuous intravenous infusion of glucose induced endogenous hyperinsulinemia and pathological changes of the lamellar tissues in standardbred horses (de Laat et al., 2012). Nonetheless, the precise mechanisms underlying both experimental and naturally occurring vascular pathology remain unclear.

Thus, appropriate screening models are required to study cellular and molecular mechanisms underlying the development of MetS-related vascular complications and to identify potential therapeutic agents. *In vitro* models serve as an important tool for studying cell behaviour under controlled conditions. To mimic vascular wall dysfunction *in vitro*, 2D cultures of primary cells present in the vascular wall (endothelial and smooth muscle cells) are mostly used (Fiorello et al., 2020; Ciechanowska et al., 2022). Other studies used 3D organoids that are capable of assembling a capillary network (Wimmer et al., 2019), or a microfluidic vessel-on-chip platform (Locatelli et al., 2022). However, these studies focused on the microcapillary network, leaving the larger blood vessels (< 6 mm in diameter), which are also affected when MetS occurs, beyond the scope of research. The ideal tissue-engineered vascular graft structure must resemble all mechanical and biological properties similar to native vessels. As such, the selection of material for scaffold manufacturing becomes crucial. Acrylate-endcapped urethane-based polymers (AUP) with a poly (ethylene glycol) (PEG) backbone (AUPPEG) are urethane-based polymers exhibiting excellent mechanical properties such as high flexibility and strength (Ionescu et al., 2021). AUPPEG is a hydrophilic polymer that exhibits fast crosslinking kinetics, making it well-suited for further processing with various 3D printing technologies (Pien et al., 2024), and its properties can be easily tuned to match the requirements of a particular application by varying the molar mass of the PEG backbone and photo-crosslinkable end groups. The biocompatibility of PEG is attributed to its low toxicity, inertness, unique physicochemical characteristics, and a well-established safety profile, with FDA-approved applications including laxatives (Lee-Robichaud et al., 2010), lubricants, and tissue sealants (Jarrett and Coury, 2013), as well as drug delivery systems (Wang et al., 2023). The use of PEG has also been described in equine veterinary medicine as a peroral administration of Kinderlax® (polyethylene glycol 4000) for the prevention of meconium retention in neonate foals (Brauch et al., 2020). However, AUPPEG does not possess cell-interactive properties.

Collagen, gelatin, and their derivatives gained increasing attention as versatile and biocompatible materials for tissue engineering applications (Klimek and Ginalska, 2020; Hu et al., 2024). These biopolymers provide a coating that differs in mechanical and structural characteristics (Maher et al., 2022). Native collagen, for example, self-assembles in triple-helical molecules and aggregates into ordered fibrils, forming a highly organised structure that closely resembles the native extracellular matrix. In contrast, gelatin and gelatin derivatives represent heat-denatured, partially hydrolyzed collagen chains. They establish a network through physical gelation or chemical crosslinking, resulting hydrogel structures. Furthermore, they facilitate cell adhesion through different integrin receptors. For instance, the linear RGD domain in gelatin and its derivatives interacts with α5β1 and αvβ3 integrins, while fibrillar collagen interacts through triple-helical ligands, such as GXOGER, with α1β1, α2β1, α10β1 and α11β1 integrins (Davidenko et al., 2016). Thus, to guarantee that the resulting scaffold architecture and bioactivity are optimally tailored to the targeted tissue environment, it is essential to select appropriate coating and processing conditions. Previous studies have demonstrated that coating synthetic polymers with collagen (Polini et al., 2010; Silva Couto et al., 2023), gelatin (Chen et al., 2021), or their derivatives (Chen et al., 2021), is essential for enhancing their cell-interactive properties. For example, a gelatin methacryloyl (GelMA) coating increases the cell-interactive properties of AUPPEG-based scaffolds towards different types of mammalian cells (Houben et al., 2016; Mignon et al., 2019; Zegwaart, 2023). However, the potential of AUPPEG scaffolds in *vitro* vascular wall models with different coatings is yet to be explored. Therefore, the present study aimed to evaluate the effect of coating AUPPEG scaffolds manufactured via digital light processing (DLP) with collagen or gelatin derivatives on their cell-interactive properties toward equine mesenchymal stromal cells (MSC) and endothelial cells (EC).

## 2 Materials and methods

**FIGURE 1:**
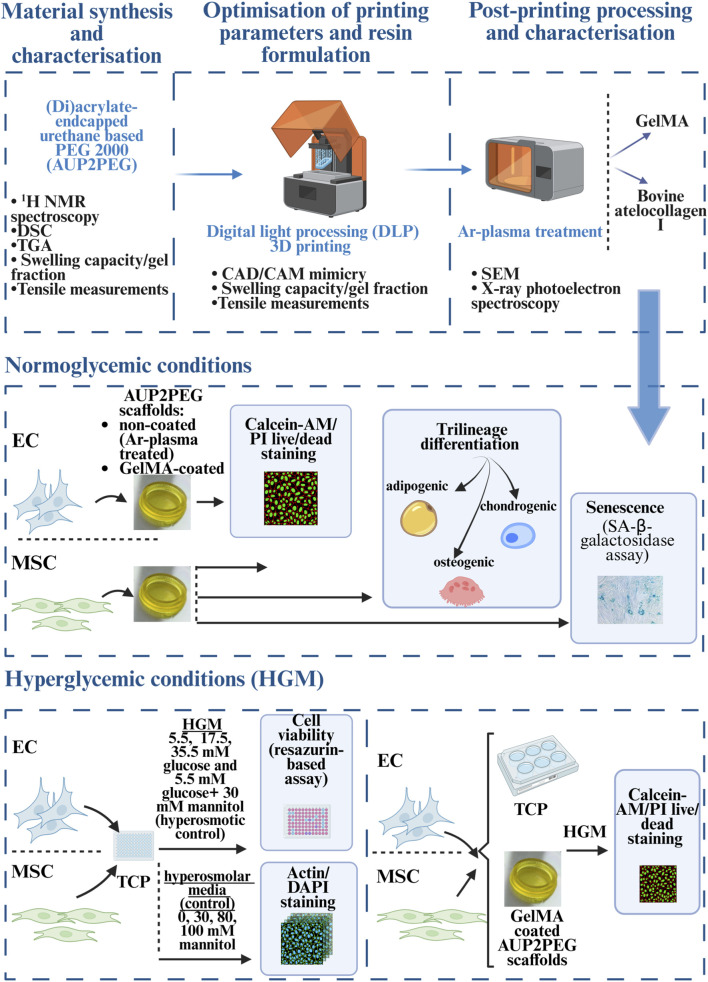
Schematic illustration of the experimental design. (TCP: tissue grade culture plastic; PI: propidium iodide; SA: senescence-associated; HGM: media with high glucose concentration). Created in http://BioRender.com.

Figure 1 provides a schematic representation of the general experimental design of the material synthesis and characterization workflow and the *in vitro* study. The constructs manufactured via DLP and coated with collagen or gelatin derivatives were characterized using several techniques, including (1) scanning electron microscopy (SEM) to assess the surface morphology, (2) mechanical testing to evaluate the mechanical properties of the DLP-processed constructs, (3) X-ray photoelectron spectroscopy to characterize the chemical composition of the scaffolds before and after coating, and (4) *in vitro* cell culture experiments to evaluate the effect of coating on EC and MSC adhesion, viability, proliferation, senescence as well as on the trilineage differentiation potential of MSC (towards adipo-, chondro- and osteogenic lineage). Moreover, the effect of hyperglycemic culture conditions, mimicking the metabolic syndrome milieu, on the EC and MSC was compared to normoglycemic culture conditions.

### 2.1 Material synthesis

#### 2.1.1 Synthesis of acrylate-endcapped urethane-based precursor with a poly(ethylene glycol) backbone (AUP2PEG)

AUP with a PEG backbone (2000 g mol^−1^) (AUP2PEG) was synthesized as described earlier (Supplementary Figure S1) (Houben et al., 2017). In brief, PEG was reacted at 65 °C in a 1:2 molar ratio with isophorone diisocyanate using 500 ppm of butylhydroxytoluene, and 300 ppm bismuth neodecanoate. In a second step, biosomer PEA-6 (MW 336 Da) was added in a 1:2 molar ratio at 80 °C. After completion of the reaction, 500 ppm of phenothiazine and 500 ppm of triphenylphosphite were added as stabilizers. After completion of the reaction, the material was poured into plates and cooled down at room temperature (RT), protected from the light.

#### 2.1.2 Synthesis of gelatin methacryloyl (GelMA)

GelMA was prepared via the reaction of the primary amines with methacrylic anhydride (MeAnH) (Supplementary Figure S3) as described in (Tytgat et al., 2019). In brief, 2.5 equivalents of MeAnH with respect to the primary amines were added to a 10% (w/v) gelatin solution in PBS (pH 7.8). The resulting mixture was stirred at 37 °C for 1 h. Then, the reaction mixture was diluted 1:1 with ultrapure water (UPW), and dialyzed (MWCO = 12–14 kDa) against distilled water for 24 h at 40 °C, followed by freeze-drying for 48 h (Christ Freeze dryer Alpha I-A).

### 2.2 Material characterization

#### 2.2.1 ^1^H NMR spectroscopy

The molecular weight of AUP2PEG and acrylate concentration were quantified via proton nuclear magnetic resonance (^1^H NMR) spectroscopy (Bruker Avance II 400 MHz) using deuterated chloroform as solvent as described earlier (Pien et al., 2024). The degree of substitution for the gelatin derivatives was quantified via ^1^H NMR spectroscopy (Bruker Avance III HD 500 MHz) using D_2_O as solvent at elevated temperature (40 °C). The calculation of the degree of substitution was performed according to (Ovsianikov et al., 2011). The ^1^H-NMR spectra for both AUP2PEG (Supplementary Figure S2) and GelMA (Supplementary Figure S4) recorded to further analyze and confirm the chemical structure of the synthesized polymer are presented in Supplementary material section.

### 2.3 Material processing and post-print processing

#### 2.3.1 Fabrication of DLP-based printed AUP2PEG scaffolds

**FIGURE 2:**
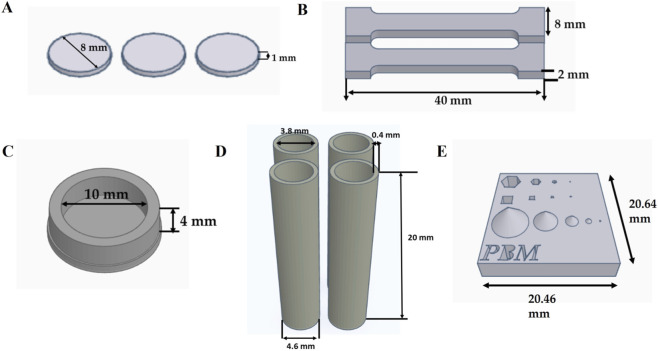
CAD models to fabricate **(A)** 2D scaffolds (discs with diameter 8 mm and thickness of 1 mm for swelling degree/gel fraction analysis); **(B)** dogbone-shaped samples for mechanical testing; **(C)** 3D well constructs with inner diameter 10 mm, a height of 4 mm and a wall thickness of 1.5 mm (for biological evaluation); **(D)** 3D tubular constructs with an inner diameter of 3.8 mm, a height of 20 mm, and a wall thickness of 400 μm; **(E)** 3D benchmark design to compare resolution achievable with different resin formulations under optimized conditions.

Constructs were fabricated using the Cell Inc. Lumen-X (Cell Inc., BICO company, United States). All resin formulations used in the study contained AUP2PEG, lithium (2,4,6-trimethylbenzoyl) phenylphosphinate (Li-TPO-L) as a photoinitiator, and tartrazine as a photoabsorber. Li-TPO-L was employed as the photoinitiator in this study owing to its higher water solubility (*<*8.5% w/v) relative to the widely utilized Irgacure 2959, its capacity to initiate polymerization under both UV-A irradiation (365 nm) and visible light exposure (405 nm, as implemented in the LumenX system), and its higher cytocompatibility (as compared to Irgacure 2959 and eosin-Y) (Elkhoury et al., 2023). UPW was used as a solvent. The resin formulation and printing conditions are presented in Supplementary Tables S2,3. The working curves for resin formulations tested were obtained by measuring the thickness of a cured layer at different cure doses (the samples were printed at 50 μm layer thickness) as described in (Pien et al., 2024). They are presented in the supporting information Supplementary Figure S6. The curing time for the final resin formulation (30% (w/v) AUP2PEG, 10 mol% Li-TPO-L, 1 mol% tartrazine) was 5 s when exploiting 23.72 mW cm^−2^ power intensity. Various structures were printed for further evaluation. An overview of the CAD models of structures selected for printing is presented in Figure 2. After the printing was finished, the samples were leached for 4 days with UPW. For the swelling degree and gel fraction determination, the samples were frozen directly after printing. To study the 3D printing process accuracy with respect to the CAD model after the DLP printing process, scaffolds were studied directly after printing by micro-computed tomography (μCT) and light microscopy. μCT images were acquired in “High resolution mode” with a preclinical X-CUBE micro-CT system (MOLECUBES; Ghent, Belgium), as described in (Muller et al., 2022). Acquired images were analysed using the Horos Project software.

### 2.4 Post-printing processing

#### 2.4.1 GelMA coating

The surface of the scaffolds was modified with a GelMA coating. Prior to the modification, the scaffolds were dried in a desiccator containing 275 g of silica for 4–5 days at 22 °C (until the mass of the scaffold was reduced to less than 20% of the wet mass). Briefly, the scaffolds were subjected to Argon plasma using a FEMTO plasma reactor version 3 (Diener Electronic, Germany) for 0.5 min at a pressure of 0.8 bar, and a power of 100 W. Next, the scaffolds were exposed to an ambient atmosphere for 20 min. To perform the coating, the plasma-activated AUP2PEG scaffolds were immersed in a 2% (w/v) GelMA (with degree of substitution (DS) 99.7%) solution in UPW containing 2 mol% Li-TPO-L at 37 °C for 18 h to allow complete rehydration of the scaffold. Next, the scaffolds were exposed to UV-A radiation (356 nm, 8 mW cm^−2^) for 60 min to induce covalent crosslinking.

#### 2.4.2 Atelocollagen coating

The surface of the scaffolds was modified with atelocollagen type I coating by physical adsorption of the protein. Before the modification, the scaffolds were dried in a desiccator and subjected to an argon plasma treatment as described in the procedure to apply the GelMA coating (*vide supra*). The coating protocol was adapted from (Polini et al., 2010). To perform the coating, the plasma-activated AUP2PEG scaffolds were immersed in a 1 mg mL^−1^ solution of bovine atelocollagen type I (isolated as described in (Zeugolis et al., 2008)) in 0.01 M acetic acid at 4 °C for 18 h, without shaking. As the formation of collagen fibrils can start from partially adsorbed collagen molecules by assembly of free segments (Dupont-Gillain, 2014), the incubation was followed by transfer of scaffolds to PBS (pH 8) and incubated at 37 °C for 60 min to induce collagen assembly.

#### 2.4.3 Sterilization

Samples were sterilized by immersion of the scaffolds in 70% (v/v) ethanol for 24 h followed by treatment with high-intensity UV-C radiation for 30 min (Greant et al., 2023).

### 2.5 Scaffold characterization

#### 2.5.1 Swelling capacity and gel fraction determination

Swelling capacity and gel fraction tests were performed on DLP-printed discs 8 mm in diameter. Directly after printing, discs were frozen at −20 °C. The samples were freeze-dried for 24 h (Christ Freeze dryer Alpha I-A). The dry mass of the samples was recorded (W_d_). Afterwards, each sample was immersed in 1 mL of UPW and incubated at 20 °C for 72 h. The wet mass of the samples was recorded at equilibrium swelling (W_s_). Then the samples were frozen at −20 °C overnight and freeze-dried again for 24 h. The dry mass of the freeze-dried samples was recorded (W_fd_). The swelling capacity (SC) (a) and gel fraction (GF)(b) were calculated using the following formulas:

$$\left(a\right)\;\text{SC}\left(g\;water/g\;material\right)=\frac{W_{s}-W_{fd}}{W_{fd}};$$

$$\left(b\right)\;\text{GF}\left(\%\right)=\frac{W_{fd}}{W_{d}}\times 100.$$

#### 2.5.2 Tensile measurements

Tensile tests were performed on a Tinius Olsen 5ST (Tinius Olsen, Ltd., United Kingdom) at 22 °C. A 500 N load cell was used for the experiments. Dogbone-shaped (40 mm long, 5 mm wide, and 2 mm thick) samples were fabricated using DLP (see the CAD model in Figure 2B) and leached out for 4 days in UPW. The samples were subjected to testing in an equilibrium swollen state. The tensile tests were performed with a preload of 0.1 N and a speed of 10 mm min^−1^ until the break. The Young’s modulus was derived from the linear slope of the stress-strain curve.

#### 2.5.3 Morphological characterization of the developed scaffolds

Microstructural characterization of the developed scaffolds was performed by scanning electron microscopy (SEM). Air dried samples of DLP-printed scaffolds were coated with an automatic Au sputter coater K550X (EmiTech) for 60 s at 15 mA under vacuum using an RV3 two-stage rotary vane pump and examined for morphological details with the JEOL JCM-7000 NeoScope benchtop scanning electron microscope (Tokyo, Japan).

#### 2.5.4 X-ray photoelectron spectroscopy (XPS)

To study the surface chemical composition, X-ray photoelectron spectroscopy (XPS) was performed using a S-Probe Monochromatized XPS spectrometer from Surface Science Instruments (VG) with the following characteristics: Source: Al K-α x-ray (1486.6 eV) monochromatic, take off angle: q = 45°, voltage and power of the source: 10 kV and 200 W respectively. A flood gun and Ni grid were used for compensation of charging effects. The XPS survey spectra were collected with a pass energy (Ep) of 140.83 eV and energy steps (Es) of 0.2 eV, the high-resolution spectra with Ep = 90.196 eV and Es = 0.1 eV. The spot size was 250 × 1000 μm^2^. The base pressure of the XPS chamber was 1 × 10^−7^ Pa. All XPS spectra were analyzed using the Casa XPS software package.

### 2.6 *In vitro* cytocompatibility assessment

#### 2.6.1 Isolation and characterization of endothelial cells

Equine EC were isolated from the vascular wall of an adult vein of a front limb, obtained *post mortem* at the local abattoir using a slightly modified protocol (De Schauwer et al., 2012; Laval et al., 2015). Briefly, after extensive washing with DPBS, cells were isolated by enzymatic digestion using collagenase type I and dispase II for 30 min at 38.5 °C in a humidified atmosphere containing 5% CO_2_. The obtained cell suspension was centrifuged (200 g, 10 min, 4 °C) and the pellet was resuspended in culture medium consisting of EGM^™^-2 supplemented with 5% FBS, 1% ABAM, and 50 μg ml^−1^ gentamycin. Cells were plated in flasks coated with 0.1% gelatin and cultured at 38.5 °C in a humidified atmosphere containing 5% CO_2_. To obtain a pure EC population, passage two (P2) cells were sorted (FACS Melody Cell Sorter, Becton-Dickinson) after incubation with 10 μg mL^−1^ acetylated low-density lipoprotein labelled with 1,1′-dioctadecyl – 3,3,3′,3′-tetramethyl-indocarbocyanine perchlorate (Dil-Ac-LDL, λ_ex_ = 545nm, λ_em_ = 585 nm) for 4 h at 38.5 °C. Subsequently, viable (Sytox Blue negative, λ_ex_ = 444nm, λ_em_ = 480 nm) Dil- Ac-LDL positive cells were either again plated in fresh culture medium or frozen upon use. Cells of passage 3-7 were used in subsequent experiments.

#### 2.6.2 Isolation and characterization of mesenchymal stromal cells

Equine MSC were isolated from subcutaneous adipose tissue collected in the local abattoir by enzymatic digestion with collagenase type I, as routinely performed in the lab (Heyman et al., 2022). Isolated cells were cultured in DMEM-LG supplemented with 30% FBS, 10^−11^ M dexamethasone, 1% ABAM, and 1% L-glutamine, at 38.5 °C and 5% CO_2_. Starting from P1, the cells were cultured in expansion medium (medium without dexamethasone) and seeded at a density of 5,000 cells·cm^−2^. Cells of passages 3-7 were used in subsequent experiments.

#### 2.6.3 Seeding of both cell types on the DLP-printed surfaces

The scaffolds were equilibrated in DMEM-LG, supplemented with 10% FBS, 1% ABAM, and 1% L-glutamine for 24 h at 4 °C. Prior to cell seeding, the scaffolds were transferred into the cell-specific culture medium. EC and MSC were plated at a density of 20 000 cells·cm^−2^ and 10,000 cells·cm^−2^, respectively, on GelMA-coated scaffolds fabricated by DLP-printing. Cells were cultured at 38.5 °C in a humidified atmosphere containing 5% CO_2_ and culture medium was refreshed twice weekly. All experiments were performed in triplicate.

#### 2.6.4 Analysis of cell viability

To assess cell viability, scaffolds seeded with cells were incubated with calcein-AM/PI solution (2 μg mL^−1^ of each fluorescent probe in DPBS) for 15 min and washed twice with DPBS. Subsequently, cells were fixed with 4% w/v paraformaldehyde (PFA) solution in DPBS for 18 h at 4 °C, and washed with DPBS. Images were captured using an inverted fluorescent microscope (Leica DMi8 Inverted microscope, Leika, Germany), and four random images per scaffold per replicate at λ_ex_ = 488, λ_em_ = 509 nm (Calcein-AM) and λ_ex_ = 548, λ_em_ = 608 nm (PI) were evaluated. Cell numbers were calculated for 3 different images per group (S_image_ = 427 μm^2^) while cell viability was calculated for 4 different images per group.

#### 2.6.5 Trilineage differentiation of MSC on the GelMA-coated AUP2PEG scaffolds

To evaluate whether the scaffolds support MSC function, trilineage differentiation (towards adipo-, chondro- and osteogenic lineage) was performed, as previously described (Heyman et al., 2022). Non-induced cells in expansion medium were used as negative controls.

*Osteogenic differentiation*. At day 4 post-seeding, osteogenic differentiation was induced by replacing the expansion medium with osteogenic differentiation medium containing DMEM-LG supplemented with 10% FBS, 0.05 mM L-ascorbic acid-2-phosphate, 10^–7^ M dexamethasone, 10 mM β-glycerophosphate, 50 μg ml^−1^ gentamycin and 1% ABAM for 11 days. Osteogenic differentiation was confirmed by Alizarine Red S staining after 18 h fixation in 4% PFA and quantification of calcium phosphate deposition (Duquesne et al., 2025). Briefly, samples were fixed overnight in 4% PFA solution at 4 °C. After washing three times in DPBS, the scaffolds were each transferred into 0.25 mL of 1 N HCl and incubated at 60 °C overnight. Before analysis, the supernatant was further diluted 1:100 in 1N HCl. Results were compared to the standards, obtained from serial dilutions of 20 μg mL^-1^ and 100 μg mL^−1^ CaCl_2_ stock solutions. After 10 min incubation with the dye at RT, absorbance was measured using the Multiskan Sky Microplate Spectrophotometer (ThermoFisher, United States) at 580 and 750 nm. All samples were run in triplicate.

*Adipogenic differentiation.* At day 4 post-seeding, adipogenic differentiation was induced by replacing the expansion medium with adipogenic induction medium containing DMEM-LG supplemented with 10^−6^ M dexamethasone, 0.5 mM 3-isobutyl-1-methylxanthine, 10 μg ml^−1^ rh-insulin, 0.2 mM indomethacin, 15% rabbit serum, 50 μg ml ^1^ gentamycin, and 1% ABAM for 72 h, followed by 24 h in adipogenic maintenance medium, which was identical to the adipogenic induction medium but lacking dexamethasone, indomethacin, and 3-isobutyl-1-methylxanthine. After two cycles, adipogenic differentiation was confirmed by Oil Red O/Mayer’s modified hematoxylin staining.

*Chondrogenic differentiation.* To induce chondrogenic differentiation, 25x10^4^ MSC were pelleted in 15 mL falcon tubes by centrifugation at 200 g for 5 min at RT and incubated for 72 h in expansion medium at 38.5 °C and 5% CO_2_. Subsequently, the cell pellet was carefully transferred onto the surface of the AUP2PEG GelMA-coated scaffold and culture medium was replaced by chondrogenic induction medium consisting of basal chondrocyte differentiation medium (Lonza), supplemented with 10 ng ml^−1^ Transforming Growth Factor-β3 for 21 days. Scaffolds were fixed in 4% PFA solution for 24 h at 4 °C, embedded into 1.5% (w/v) low-melting point agarose on DPBS, and further processed for paraffin sectioning (slices of 5 μm). Alcian blue staining with Nuclear Fast Red counterstaining was performed to confirm chondrogenic differentiation.

#### 2.6.6 Analysis of senescence using ß-galactosidase assay

Senescence-associated ß-galactosidase activity was detected according to (Heyman et al., 2025). Briefly, MSC were seeded on the scaffolds, with a density of 5 250 cells·cm^−2^ and TCP was included as control group. Cells were cultured for 5 days in expansion medium at 38.5 °C in a humidified atmosphere containing 5% CO_2_ before the assay was performed.

### 2.7 Cell culture in hyperglycemic conditions

#### 2.7.1 Effect on cell viability in 2D cultures

Models that mimic diabetic conditions *in vitro* usually use glucose concentrations substantially higher than those observed *in vivo*. In humans, hyperglycemia is defined as blood glucose level higher than 7 mM, and in equine patients, glucose levels exceeding 7.3 mM are considered hyperglycemic. In contrast, in *vitro* studies using human cells, media containing 25–50 mM glucose or even higher are often used (Mascardo, 1988; Busik et al., 2008; Uruski et al., 2021; Wang et al., 2021). However, to best our knowledge, there are no data published describing the influence of high glucose medium on the viability of equine endothelial and mesenchymal stromal cells *in vitro*. In this study, EC and MSC were cultured in the media containing 17,5, and 35,5 mM as high glucose media. For the study, EC and MSC were seeded in 96-well plates with seeding densities of 18750 and 12500·cells·cm^−2^, respectively, in their respective media (containing 5.5 mM glucose) for 24 h at 38.5 °C and 5% CO_2_, before switching to hyperglycemic conditions. Two increased glucose concentrations were tested, namely, 17.5 and 35.5 mM glucose. Viability was assessed using the resazurin assay. It should be mentioned that the addition of a high concentration of glucose to the culture medium increases its osmolarity to levels above the physiological range. HGM triggers the aldose reductase pathway and results in increased intracellular sorbitol concentrations, which counteract the extracellular osmotic pressure (Lewko et al., 2011). The latter does not occur when cells are cultured in the presence of high mannitol concentrations, which is used to equalize the osmotic pressure on the cells (Ciechanowska et al., 2022), resulting in prolonged hyperosmotic stress. Therefore, an osmotic control was included by adding mannitol to adjust osmolarity. Following groups were included: 5.5 mM glucose without mannitol (normoglycemic, iso-osmotic control); 5.5 mM glucose +30 mM mannitol (normoglycemic, hyperosmotic control); 17.5 mM glucose +18 mM mannitol (hyperglycemic (HGM), hyperosmotic); 35.5 mM glucose (hyperglycemic (HGM), hyperosmotic). After 24 h and 48 h of incubation, media were replaced with fresh media containing 50 μM resazurin. 24 h later, 150 μL of the resulting cell-conditioned medium was transferred into a Thermo Scientific™ Nunc 96-Well Flat Bottom plate (ThermoScientific, United States) to measure the medium’s fluorescence using an Infinite M200 PRO microplate reader (TECAN, Switzerland) with excitation wavelength (λEx) at 560 nm and emission wavelength (λEm) at 590 nm.

As it has been reported that hyperosmotic culture conditions induce long-term morphological changes in human MSC (Potočar et al., 2016), we also assessed MSC morphology in mannitol-containing media. To this end, MSC were seeded in a 12-well plate with a density of 10,000 cells·cm^-2^ in expansion medium. After 18 h of incubation at 38.5 °C and 5% CO_2_, the medium was changed to expansion medium supplemented with different mannitol concentrations (0, 30, 80, 100 mM, with medium osmolarity ranging from 320 to 428.5 mOsm·L^−1^) for 48 h. Subsequently, cells were fixed with 4% w/v PFA for 15 min at RT, and permeabilized with 0.1% Triton-X-100 in DPBS for 15 min at RT. Upon permeabilization, the cells were washed twice with DPBS. The F-actin was stained with ActinGreen™ 488 ReadyProbes® Reagent, while the nuclei were counterstained with 4′,6-diamidino-2-phenylindole (DAPI). Cells were imaged using an inverted fluorescent microscope (Leica DMi8 Inverted microscope, Leika, Germany). Four random images per cultivation condition per replicate at λ_ex_ = 495, λ_em_ = 519 nm (FITC) and λ_ex_ = 353, λ_em_ = 465 nm (DAPI) were evaluated. The nuclear area was determined from obtained digital images using FIJI (ImageJ2). The nuclear area was calculated for at least 100 cells from each group.

#### 2.7.2 Effect on cell viability when seeded on GelMA-coated AUP2PEG scaffolds

24 h after EC and MSC, respectively, were seeded on GelMA-coated AUP2PEG scaffolds, medium was replaced with medium containing 35.5 mM glucose (hyperglycemic, hyperosmotic). After 72 h of incubation at 38.5 °C and 5% CO_2_, the calcein-AM/PI staining was performed to identify living/dead cells. Viability was compared with control cells cultured in normoglycemic hyperosmotic conditions (5.5 mM glucose +30 mM mannitol) and in normoglycemic, iso-osmotic conditions (5.5 mM glucose without mannitol).

### 2.8 Statistical analysis

Statistical analysis was performed using GraphPad Prism 10 (Domatics, United States). The normality of the data was assessed by visual inspection of the Q-Q plots, and equality of variances was assessed by plotting the residuals against the fitted values. Unpaired *t*-test, one-way or two-way ANOVA was used to analyse data that followed a normal distribution. For data not following a normal distribution, the Mann-Whitney U test was used. The statistical tests and the number of replicates are indicated at the bottom of the figures and tables in the manuscript. The values are expressed as mean ± SD. Differences with p < 0.05 were considered significant.

## 3 Results and discussion

### 3.1 Optimization of AUP2PEG-based resin formulation for the DLP

It is known that the stiffness of a cross-linked hydrogel directly correlates with the polymer concentration (Schittecatte et al., 2023), and it is possible, to a certain extent, to tune the mechanical properties of the scaffold by modifying not the polymer itself but rather by changing the resin formulation and printing conditions. As previously reported, AUP2PEG polymers can be used to efficiently manufacture different types of scaffolds using DLP 3D printing (Pien et al., 2024). This is a light-induced additive manufacturing technique that enables the rapid reconstruction of various structures with high resolution (XY resolution down to 35 μm and Z resolution down to 50 μm) and intricate details (Pien et al., 2024). Thus, we tested the processability of the resin containing 30% (w/v) of AUP2PEG using DLP 3D printing aiming at manufacturing scaffolds mimicking relatively soft tissues (with Young’s moduli situated between 0.5 and 1.5 MPa, for example, small blood vessels (e.g., human common carotid artery: 0.396 ± 0.12 MPa to 0.90 ± 0.25 MPa (Khamdaeng et al., 2012; Szeghy et al., 2022)), and equine aorta–approximately 0.9 MPa (Bowser et al., 2014)).

To achieve this goal, we have tested different resin formulations (detailed description is provided in the Supplementary Material section) and selected a resin formulation comprised of 30% (w/v) AUP2PEG, 10 mol% Li-TPO-L, 1 mol% of tartrazine dissolved in UPW. The optimized printing parameters were as follows: an irradiation time of 5 s, and a light intensity of 23.72 mW cm^−2^. This resin formulation demonstrated good printing repeatability, and printed scaffolds exhibited good CAD/CAM mimicry not only directly after printing, but also exhibiting the smallest changes of the scaffold geometry during further processing. The study of physico-chemical properties of the scaffolds revealed that samples manufactured with the selected resin demonstrated a high gel fraction (89.26% ± 0.89%), indicating efficient crosslinking of the polymer network. The swelling capacity of scaffolds was 3.8 ± 0.05 g_water_/g_material_. Next, the mechanical properties of the scaffolds manufactured using the resin formulation were tested using tensile testing of the dogbone-shaped scaffolds (Figure 2; Figure S8). Published biomechanical data show that the Young’s modulus of human small arteries lies approximately between 0.40 and 0.90 MPa (e.g., human common carotid artery under physiological loads) (Khamdaeng et al., 2012; Szeghy et al., 2022). The mechanical properties of our DLP-printed constructs (Young’s modulus 0.6 ± 0.1 MPa, ultimate force 2.08 ± 0.60 N, maximum stress 0.227 ± 0.06 MPa, elongation 47.0% ± 14.9%) therefore fall within the lower physiological range reported for human small arteries. Comparable equine data for vessels <6 mm in diameter are not yet available, but published values for major equine arteries indicate, for example, an equine aortic Young’s modulus of ≈0.9 MPa, placing our constructs close to the species-relevant physiological stiffness spectrum (Bowser et al., 2014). While earlier studies on AUP2PEG did not address vascular models specifically, they demonstrated that the stiffness of AUP2PEG-based scaffolds can be tuned by increasing polymer concentration, reaching moduli up to 1.89 ± 0.076 MPa (Pien et al., 2024). This tunability allows matching scaffold stiffness to the requirements of different vascular applications.

### 3.2 Characterisation of DLP-printed tubular constructs

**FIGURE 3:**
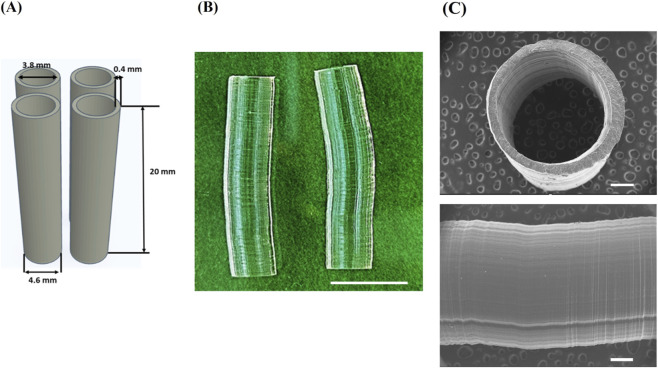
DLP-printed tubular constructs. **(A)** CAD model used for the fabrication of 3D tubular constructs with an inner diameter of 3.8 mm, a height of 20 mm, and a wall thickness of 400 μm; **(B)** DLP 3D printed tubular scaffolds from AUP2PEG after leaching in UPW for 72 h. Scale bar represents 1 cm; **(C)** Scanning electron micrographs (SEM) of the tubular scaffold. The scale bar represents 500 µm.

Several studies have highlighted the importance of mechanical stimulation during the *in vitro* maturation of vascular tissue (Devillard and Marquette, 2021). As a result, *in vitro* vascular perfusion systems, referred to as “vascular bioreactors”, are becoming increasingly significant as they effectively integrate 3D vascular cell cultures with relevant physical and hemodynamic forces (Mitchell et al., 2023). Nevertheless, the utilization of a bioreactor requires the consideration of specific criteria regarding the minimal length of the tubular constructs that can be effectively mounted within the device. In vascular bioreactors, the standard length of the construct employed typically falls within a range of 1–5 cm. For example, in the Easy Flow bioreactor system, a construct with a length of 2–4 cm can be efficiently mounted (Matos et al., 2022). Therefore, we evaluated the possibility of manufacturing 2 cm-long tubular constructs that could serve as a scaffold for the subsequent development of an *in vitro* cellularized model of the vascular wall using the selected resin formulation (Figure 3A,B).

After printing and subsequent swelling in UPW for 72 h, the inner diameter, wall thickness, and length of the tubular scaffolds were quantified. Upon swelling to equilibrium, the constructs displayed dimensional deviations of +13.2% in length (CAD/CAD fidelity 113.2%), +11.7% in outer diameter (CAD/CAD fidelity 111.7%), and −13.5% in wall thickness (CAD/CAD fidelity 86.5%). These values are situated within the ±15% tolerance commonly considered acceptable for hydrated polymeric vascular scaffolds intended for perfusion bioreactors, where reproducible mounting geometry and physiological flow behavior - rather than exact anatomical replication - are the primary requirements (Mitchell et al., 2023; Nguyen et al., 2023; Garciamendez-Mijares et al., 2024). The observed dimensional changes are attributable to the relatively low acrylate content and high hydrophilicity of AUP2PEG, resulting in pronounced water uptake and volumetric swelling. This swelling behavior was further confirmed in DLP-printed disk-shaped scaffolds produced from the same resin formulation. These disks showed high CAD/CAM fidelity immediately after printing (101.83% ± 0.75% in the x–y plane, 97.78% ± 8.64% in the z-direction) but increased to 111.7% ± 1.54% (x–y) and 111.25% ± 9.98% (z) after equilibrium swelling (Supplementary Figure S7A,B). Together, these findings indicate that the swelling characteristics of AUP2PEG-based constructs are reproducible across geometries and should be taken into account during design. Accordingly, the initial CAD models can be systematically pre-compensated to achieve higher post-swelling dimensional accuracy in future prints.

SEM imaging revealed circumferential layers resulting from the layer-by-layer DLP printing process (Figure 3C). Although the present study focused on endothelial cells and mesenchymal stromal cells, these circumferential surface features may still hold relevance for future multilayer vascular wall models, as numerous studies have shown that circumferential or groove-oriented micro- and mesoscale topographies can direct vascular smooth muscle cell alignment, elongation, and contractile phenotype organization - key attributes of the *tunica media* (Hsiao et al., 2015; Sun et al., 2020; Nagayama and Wataya, 2024). ECs likewise exhibit contact-guidance responses to directional topographies, including alignment, elongation, and cytoskeletal reorganization along microgroove orientations (Sprague et al., 2012; Sun et al., 2017; Fernández-Francos et al., 2021). While we did not assess SMCs in the current work, these findings suggest that the circumferential patterning inherent to DLP printing may support controlled organization of vascular cell layers in future studies designed to model the medial architecture of blood vessels.

### 3.3 Post-print functionalization: surface coating of the AUP2PEG scaffolds with gelatin and collagen derivatives

#### 3.3.1 Morphology and chemical compositions of the surface of AUP2PEG-based scaffolds

**FIGURE 4:**
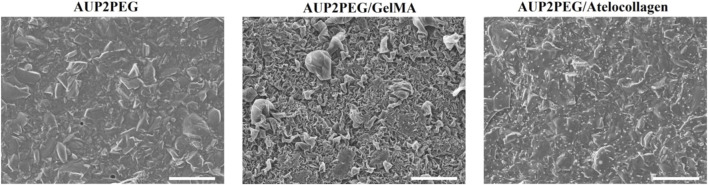
Scanning electron micrographs (SEM) of DLP-printed AUP2PEG scaffolds before and after surface modification with GelMA (AUP2PEG/GelMA) or atelocollagen I (AUP2PEG/atelocollagen). The scale bars represent 100 µm.

To promote cell attachment to the AUP2PEG-based DLP-printed constructs, we modified the surface of the developed scaffolds with GelMA or bovine type I atelocollagen. The two approaches used for coating were UV-induced photopolymerization of GelMA on the surface of the DLP-printed scaffold and physisorption of bovine atelocollagen. The latter is the widely used method to immobilize proteins on the surface, which provides better preservation of the protein’s natural configuration (Polini et al., 2010). The surface morphology of the scaffolds before and after surface modification was investigated through SEM. Analysis of the scaffolds after surface modification revealed the changes in surface morphology. On the surface of the AUP2PEG scaffolds coated with GelMA, we observed a thin film forming folded structures on the surface of the scaffold (Figure 4). The surface of the AUP2PEG scaffolds modified with atelocollagen exhibited only minor, punctate structures that were unevenly distributed across the surface.

XPS elemental surface analysis was performed to characterize GelMA, atelocollagen-coated, and non-coated AUP2PEG-based scaffolds manufactured by DLP. The data obtained from the XPS analysis (Table 1), together with the SEM-based surface morphology evaluation, indicate that physisorption of bovine atelocollagen resulted in only minimal protein deposition on the surface of the AUP2PEG DLP-printed scaffolds, especially when compared to the substantially higher nitrogen signal obtained for the GelMA-coated samples. To the best of our knowledge, no prior reports have examined atelocollagen physisorption specifically on AUP2PEG; however, previously published XPS data for type I collagen coatings on other synthetic polymers, such as poly (L-lactide-co-glycolide) or polystyrene, show higher nitrogen atomic percentages (≈5.2–8.2%, N/C ≈ 0.076–0.10), indicating comparatively greater collagen deposition under those conditions (Adamczak et al., 2011). Literature shows that collagen deposition can be increased through chemical crosslinking, yet such approaches frequently compromise collagen’s native functionality: crosslinkers may disrupt the triple-helical structure, alter fibrillar organization, or mask bioactive sites required for integrin-mediated adhesion (Davidenko et al., 2016; Maher et al., 2022). For example, glutaraldehyde (GTA), although effective at crosslinking collagen, can leave cytotoxic aldehyde residues and has been linked to calcification (Davidenko et al., 2015). The commonly used 1-ethyl-3-(3-dimethylaminopropyl-carbodiimide hydrochloride/N-hydroxy-succinimide (EDC/NHS) zero-length crosslinking system avoids GTA-associated cytotoxicity but has been shown to reduce accessibility of collagen’s GXOGER motifs, thereby impairing integrin recognition (Bax et al., 2017). Given these drawbacks, physisorption was initially selected to preserve the native conformation of atelocollagen. However, because the atelocollagen coating achieved by physisorption was sparse and non-uniform on AUP2PEG, future work should focus on non-denaturing immobilization strategies capable of increasing collagen deposition while maintaining the integrin-binding domains characteristic of native type I collagen. In contrast, although GelMA does not retain the native triple-helical collagen structure and therefore lacks GXOGER motifs, its methacrylated backbone supports efficient photocrosslinking, tunable mechanical properties, and coating uniformity. GelMA also retains linear RGD-type adhesion sequences, which can engage α5β1 and αvβ3 integrins and thereby support cell attachment. Previous studies have reported improved human MSC and mouse calvaria preosteoblast cells adhesion on GelMA-modified AUPPEG scaffolds fabricated by electrospinning, fused deposition modeling and two-photon polymerization (Houben et al., 2017). Given these advantages and the reliable coating performance observed in our XPS and SEM analyses, subsequent biological experiments in this study were performed exclusively using GelMA-coated AUP2PEG scaffolds to fully assess their potential in an *in vitro* vascular wall model.

**TABLE 1:** Chemical composition of the modified AUP2PEG surfaces, determined by XPS analysis.

N	Sample	Elemental composition (%)			N/C ratio
		C 1s	N 1s	O 1s	
1	AUP2PEG	77.01 ± 2.58	-----	22.99 ± 2.58	------
2	AUP2PEG/GelMA	75.96 ± 4.29	4.06 ± 0.85^a^	19.98 ± 4.56	0.053
3	AUP2PEG/Bovine typeI atelocollagen	71.94 ± 3.36	0.67 ± 0.09^a^	27.39 ± 3.45	0.009
4	Bovine type I atelocollagen	84.97 ± 1.62	3.57 ± 0.81	11.45 ± 0.97	0.042

^a^ The relative level of protein deposition on the scaffold surface was quantified using the N1s signal as an indicator of nitrogen-containing peptide bonds. Nitrogen atomic percentages from survey spectra acquired under identical XPS, settings were statistically compared between GelMA- and, bovine atelocollagen-coated samples. Data were analyzed using Welch’s t-test; *p < 0.05 (n = 3)*.

#### 3.3.2 *In vitro* cytocompatibility assessment

**FIGURE 5:**
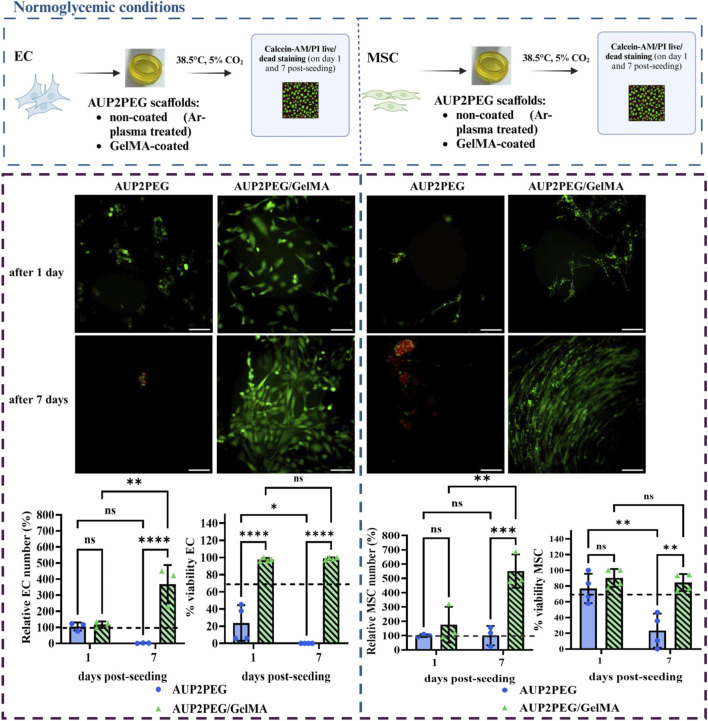
*In vitro* cytocompatibility assessment of EC and MSC cultured on DLP-printed AUP2PEG scaffolds with or without GelMA coating. Viability was evaluated using staining with calcein AM/PI on days 1 and 7 post-seeding. Images are merged for viable (green) and dead (red) cells. Scale bar represents 200 µm. According to the ISO standard (ISO 10993–5), viability above 70% is required (cut-off indicated on the graphs). Data was analyzed by two-way ANOVA with Fisher’s LSD *post hoc* test. **- p < 0.05, ***- p < 0.001; ****- p < 0.0001. A schematic illustration of the experimental design was created in http://BioRender.com.

We selected EC isolated from adult vein and MSC isolated from adipose tissue as cell types to study the effect of surface modification on cell attachment and growth, as EC represent one of the major cell types present in all blood vessels and MSCs are known to reside perivascularly and are able to differentiate into smooth muscle-like cells (Kim et al., 2009; Zhang et al., 2017). One day post-seeding, the number of cells attached to the non-coated and coated scaffolds was not significantly different for both cell types (Figure 5). To quantify the relative number of EC and MSC, a mean cell number was normalized towards the number of cells on the AUP2PEG scaffold on day 1 post-seeding (100%). At this time point, MSC demonstrated a high viability regardless of the coating (76.97 ± 16,16% in the non-coated versus 90.3% ± 9.9% in the GelMA-coated group). In contrast, only 23.67% ± 17.9% of EC remained viable on the non-coated AUP2PEG scaffold, whereas EC on the GelMA-coated counterparts exhibited an excellent viability (approximately 97.51% ± 1.64% of viable cells). These EC also displayed a more elongated morphology.

On day 7 post-seeding, the number of cells attached to GelMA-coated scaffolds was significantly higher than on the non-coated samples for both cell types. For EC, an increase to 368.6% ± 112.2% was observed, while for MSC an increase to 550.7 %± 116.9% was noted, with the number of cells on non-coated samples on day 1 post-seeding taken as a reference (they were assigned a value of 100%) (Figure 5). Additionally, a significant increase in cell number compared to day 1 was observed for both EC and MSC on GelMA-coated scaffolds (from 118.7% ± 18.5% to 368.7% ± 119.2% for EC and from 176.7% ± 125% to 550.7% ± 116.9% for MSC). The viability on GelMA-coated samples remained high (98.92% ± 1.13% for EC and 84.4% ± 9.3% for MSC), whereas in the non-coated samples, only dead (PI positive) EC were detected and viability of MSC dropped to 23.3% ± 18.6%.

Furthermore, EC exhibited their characteristic cobblestone and MSC their characteristic elongated spindle-shaped morphology on the GelMA-coated scaffolds. In conclusion, GelMA-coating of the AUP2PEG scaffolds supported the proliferation and morphology of both EC and MSCs and significantly increased cell survival during a 7-day culture period.

#### 3.3.3 Trilineage differentiation potential of MSC on GelMA-coated AUP2PEG scaffolds

**FIGURE 6:**
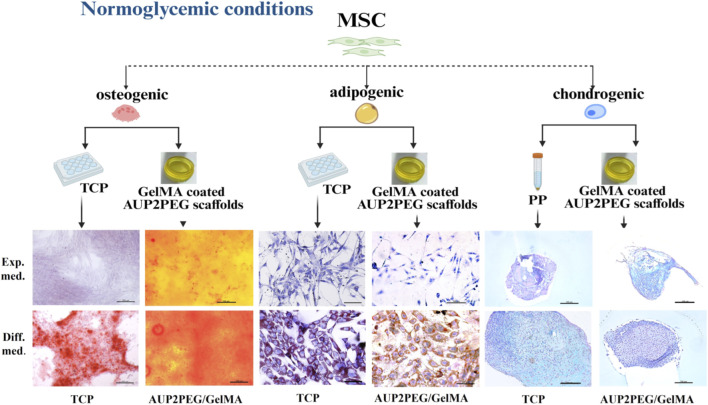
Trilineage differentiation potential of MSCs cultured on DLP-printed scaffolds coated with GelMA in either expansion (negative control) or differentiation medium. Adipogenic differentiation was confirmed using Oil Red O and Hematoxylin staining (scale bar represents 100 µm); osteogenic differentiation using Alizarin Red staining (scale bar represents 200 µm); chondrogenic differentiation using the Alcian Blue and Nuclear Fast Red staining (scale bar represents 100 µm). (TCP: tissue grade culture plastic; PP: polypropylene; exp.med.: expansion medium; diff.med.: differentiation medium). A schematic illustration of the experimental design was created in http://BioRender.com.

Since the microenvironment, in particular scaffold properties (such as composition, topography, and mechanical properties), can strongly affect cell function (Acevedo-Acevedo and Crone, 2015; Liu et al., 2022), we performed trilineage differentiation of MSC cultured on GelMA-coated AUP2PEG scaffolds. Regarding adipogenic differentiation, lipid droplets were observed after 8 days in adipogenic media, while MSCs in expansion medium remained undifferentiated (Figure 6). Regarding osteogenic differentiation, calcium phosphate crystal deposition was significantly increased after 11 days when cells were cultured in osteogenic medium, compared to MSCs cultured in expansion medium (80.28 ± 1.86 μg per sample and 55.59 ± 5.2 μg per sample, respectively, p = 0.0029, n = 3). Regarding chondrogenic differentiation, we showed that the scaffolds allowed the characteristic accumulation of sulfated glycosaminoglycans (sGAGs) after 21 days of differentiation. The micromass culture in the expansion medium also demonstrated the deposition of sGAGs, indicative for spontaneous differentiation (Heyman et al., 2022), but the pellet was smaller in size and demonstrated signs of degradation. In conclusion, GelMA-coated AUP2PEG scaffolds adequately support MSC function which, to the best of our knowledge, has not been demonstrated yet.

#### 3.3.4 The effect of GelMA-coated AUP2PEG scaffolds on cellular senescence

**FIGURE 7:**
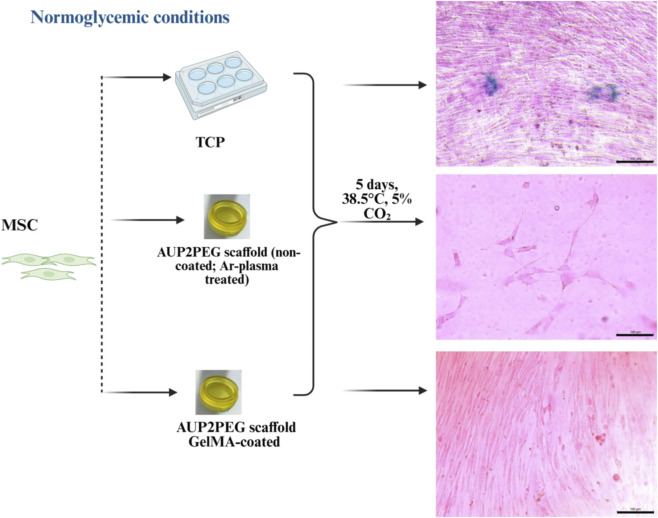
Expression of senescence-associated ß-galactosidase by MSC seeded on TCP versus non-coated and GelMA-coated DLP-printed AUP2PEG scaffolds 5 days after seeding. Seeding density in all groups was 5 250 cells·cm^-2^. Staining: 5-bromo-4-chloro-3-indolyl-β-D-galactopyranoside (X-gal) + nuclear fast red. Scale bar represents 100 µm. A schematic illustration of the experimental design was created in http://BioRender.com.

Since scaffold properties can also strongly affect cell fate (Blokland et al., 2022), we studied the effect of GelMA-coated AUP2PEG on senescence induction in MSC. When MSCs were cultured on TCP, senescence-associated ß-galactosidase-positive cells were observed 5 days post-seeding (Figure 7), whereas no ß-galactosidase-positive cells were detected when cultured on the AUP2PEG scaffolds with or without coating. This difference in senescent phenotype between AUP2PEG and TCP could be attributed to the different stiffness of both materials. AUP2PEG exhibits a stiffness of 0.738 MPa in contrast to TCP, which displays a stiffness of 2.28–3.28 GPa. It is reported that cultivation of primary fibroblasts on the GelMA with higher stiffness activated genes and secreted factors that are part of the senescence-associated secretory phenotype (SASP), as compared to the softer one (Blokland et al., 2022). Moreover, the cultivation of human MSC on the hydrogels manufactured from styrene-derived gelatin, can delay senescence progression in late passage MSC as compared to cultivation on tissue culture grade plastic coated by the same modified gelatin (Kuboki and Kidoaki, 2025).

#### 3.3.5 Effect of hyperglycemic culture conditions on EC and MSC

**FIGURE 8:**
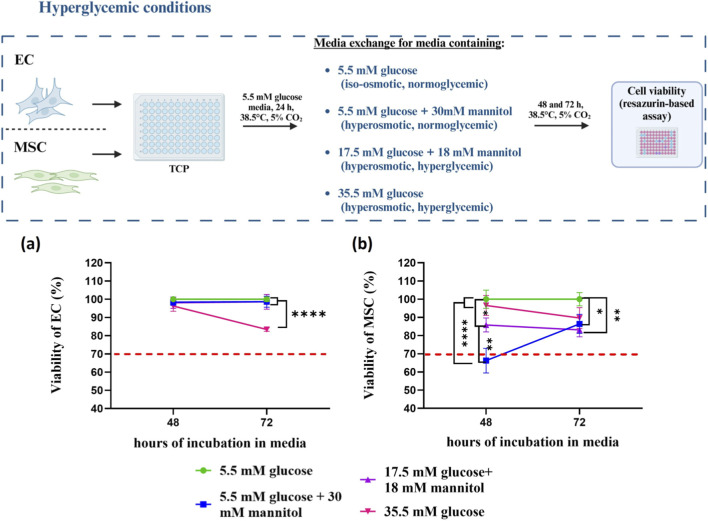
Influence of high glucose and mannitol concentrations on the viability of EC **(a)** and MSC **(b)** cultured on TCP. Data is expressed as mean ± SD (n = 3). Data were analyzed by two-way ANOVA with Tukey’s *post hoc* analysis. *p < 0.05, **p < 0.005, ****p < 0.0001- represents significant differences. A schematic illustration of the experimental design was created in http://BioRender.com.

To mimic a metabolic syndrome-like milieu *in vitro*, glucose concentrations exceeding 10 mM are used in the culture medium (Nikolits et al., 2021). However, elevated glucose concentrations (20–30 mM) might significantly impair cellular functions, e.g., inducing apoptosis, as previously reported for HUVECs (Ho et al., 2000; Hou et al., 2015; Faridvand et al., 2022). For MSC, glucose concentration in the medium is one of the most significant limiting factors for *in vitro* MSC culture given their glycolytic phenotype (Nuschke et al., 2016). The impact of hyperglycemic culture conditions on MSCs is therefore highly variable, depending on both the metabolic activity and the source of the cells. Observed effects ranged from the suppression of proliferation, induction of premature cellular senescence, and the upregulation of autophagy (Nuschke et al., 2016) to high resistance of the cells to high glucose toxicity (Al-Qarakhli et al., 2019). So far, the effect of hyperglycemic *in vitro* culture conditions on equine EC and MSC viability has not been reported yet. To study the effect of high glucose culture conditions on the viability of EC cultured in TCP, the resazurin assay was performed on EC cultured in medium with different glucose concentrations (5.5, 17.5, and 35.5 mM). Since the addition of 30 mM glucose increased the osmolarity of the culture medium from 320 to 352.6 mOsm·L^−1^, mannitol was used as an osmotic control. No significant differences were observed in the viability of EC cultured in medium containing 5.5 mM glucose (normoglycemic, iso-osmotic control) or 5.5 mM glucose +30 mM mannitol (normoglycemic, hyperosmotic control) at both time points (48 and 72 h). The viability of EC cultured in medium containing 17.5 mM glucose was not significantly different from that of the cells in both normoglycemic control groups. However, EC viability was significantly reduced after 72 h in medium containing 35.5 mM glucose (hyperglycemic, hyperosmotic group) compared to 5.5 mM glucose at the same time point (19% reduction) (Figure 8a). In literature, similar findings have been reported for HUVECs (Hou et al., 2015; Faridvand et al., 2022), although the decrease is less pronounced. On the contrary, no significant impact on MSC viability following culture in medium containing 35.5 mM glucose was observed when compared to 5.5 mM glucose (Figure 8b). Surprisingly, MSC viability in medium containing 5.5 mM glucose +30 mM mannitol (normoglycemic, hyperosmotic control) for 48 h was significantly reduced below 70% when compared to culture in both high (35.5 mM) and low glucose (5.5 mM) medium. In the 72-h cultures, viability of MSC was restored although it remained lower than the viability observed in the normoglycemic iso-osmotic control group (5.5 mM glucose). MSC cultured in medium containing 17.5 mM glucose +18 mM mannitol exhibited reduced viability at both time points although the reduction at 48 h was less pronounced compared to normoglycemic, hyperosmotic control (5.5 mM glucose +30 mM mannitol) (approximately 14% at 48 h and 16.8% at 72 h).

**FIGURE 9:**
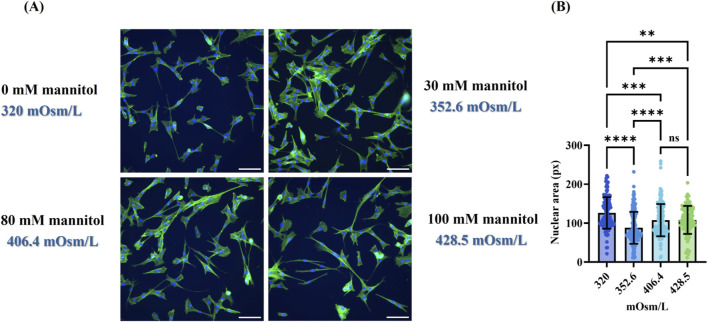
Influence of mannitol on MSC morphology cultured on TCP. **(A)** Morphology of MSC after exposure to different concentrations of mannitol (0, 30, 80, and 100 mM). Actin (green) and DAPI (blue) stained images of MSC cultured for 48 h. The scale bar represents 200 µm. **(B)** By comparing the nuclear area of at least 100 cells from different experimental groups to the control group (without mannitol), a decline in nucleus area was observed following 48 h of elevated osmolarity levels. The data was analyzed using the one-way ANOVA followed by Tukey’s multiple comparison tests, compared to the iso-osmotic group (5.5 mM glucose, 320 mOsm·L^−1^). **p < 0.005; ***p < 0.001; ****p < 0.0001.

Although mannitol is known as a non-metabolized sugar alcohol, widely used in clinical practice as hyperosmolar therapy, it has previously been shown *in vitro* that high concentrations (100–400 mM) can induce human renal tubular epithelial cell injury (Shi et al., 2018) while apoptosis was observed in bovine aortic EC (Malek et al., 1998). In the present study, however, the concentrations used were below the levels associated with adverse effects. Supplementing 30 mM mannitol increased the osmolarity of the culture medium from 320 to 352.6 mOsm·L^−1^. It has been demonstrated that long-term morphological changes have been induced in human adipose tissue-derived MSC cultured in media with osmolarities ranging from 400–600 mOsm·L^−1^, including vesicle formation, increased cell surface, and actin reorganization (Potočar et al., 2016). Furthermore, it has been reported that hyperosmotic stress resulted in shrinkage of the nucleus and chromatin condensation, which in turn caused non-homogenous DAPI staining of the nuclei (Finan and Guilak, 2010; Olins et al., 2020). In our study, no significant changes in size and spreading of the cells was observed (Figure 9A). Despite the homogeneous DAPI staining in all samples, indicating the absence of increased chromatin condensation, a significant reduction in the nuclear area however was evident in cells from all experimental groups when compared to cells cultured in normoglycemic iso-osmolar medium (5 mM glucose) (Figure 9B). Surprisingly, the highest shrinkage was observed in cells cultured in the normoglycemic, hyperosmotic group (5.5 mM glucose+30 mM mannitol).

**FIGURE 10:**
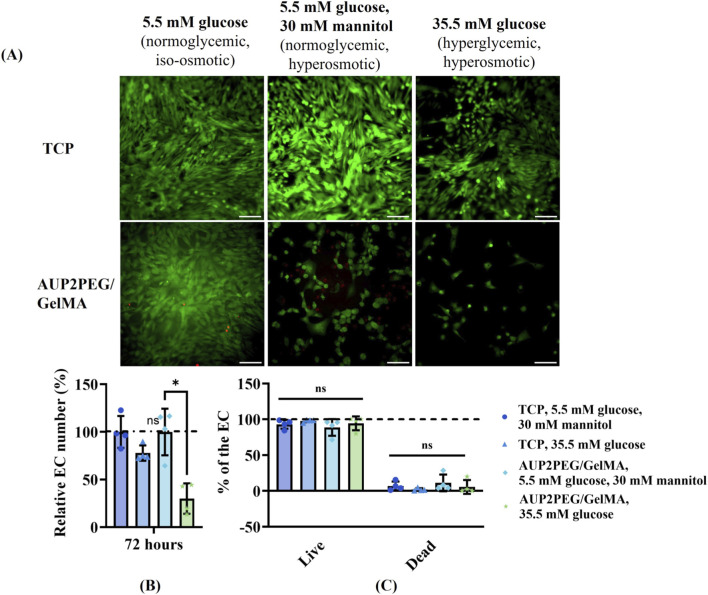
**(A)** Live/dead fluorescent staining with calcein AM/PI of EC cultured on GelMA coated AUP2PEG scaffolds in hyperglycemic conditions. Images are merged for live (green) and dead (red) cells. Scale bar represents 200 µm. **(B)** Relative cell number is showed with the mean cell number normalized towards the number of cells cultured on the same surface type under normoglycemic, hyperosmotic culture conditions (5.5 mM glucose+ 30 mM mannitol); **(C)** Quantification of live and dead cells after 72 h. Data is analyzed by two-way ANOVA with Fisher’s LSD *post hoc* test. *- p < 0.05.

**FIGURE 11:**
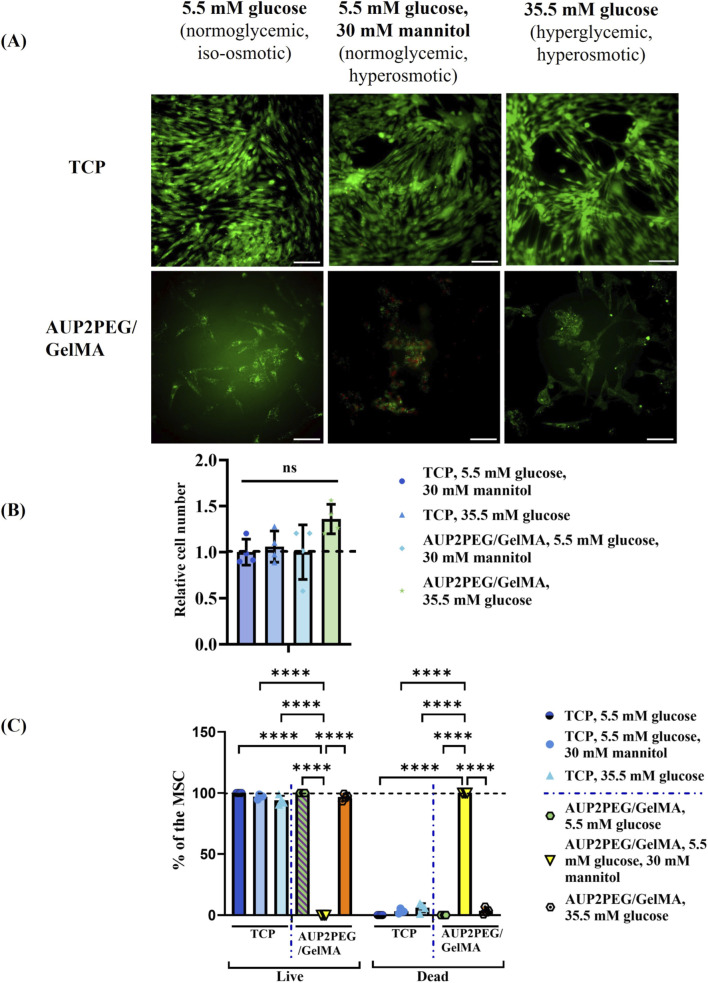
**(A)** Live/dead fluorescent staining with calcein AM/PI of MSC cultured on GelMA coated AUP2PEG scaffolds in hyperglycemic conditions. Images were merged for live (green) and dead (red) cells. Scale bar represents 200 µm. **(B)** Relative cell number is showed with the mean cell number normalized towards the number of cells cultured on the same surface type of surface and in the same culture conditions. **(C)** Quantification of live and dead cells after 72 h. Data was analyzed by two-way ANOVA with Fisher’s LSD *post hoc* test. ****- p < 0.001.

Next, we evaluated the effect of hyperglycemic conditions on the viability of EC and MSC cultured on GelMA coated AUP2PEG scaffolds. After 72 h, significantly less EC were attached to the surface in the hyperglycemic, hyperosmotic group (35.5 mM glucose) compared to the normoglycemic hyperosmotic control group (5.5 mM glucose+30 mM mannitol), i.e. 30% ± 15.93% and 99.9% ± 24.42%, respectively (Figure 10). The same trend of an increasing number of dead cells in mannitol-containing medium was observed when cells were cultured on TCP, although less pronounced, i.e. 77.8% ± 8% in the hyperglycemic hyperosmotic group (35.5 mM glucose) versus 99.9% ± 16.7% in the normoglycemic hyperosmotic control group (5.5 mM glucose+30 mM mannitol). Unexpectedly, the total cell number and number of viable cells attached to the scaffold were significantly different between the normoglycemic iso-osmotic control (5.5 mM glucose medium) and the normoglycemic hyperosmotic control (5.5 mM glucose +30 mM mannitol), suggesting that cells cultured on a scaffold become more sensitive to increased osmotic pressure than cells cultured on TCP. In order to make sure that the negative control group adequately mimics the *in vivo* situation of healthy animals, the effect of mannitol on the viability of cells cultured in 3D conditions should be further explored, or L-glucose can be alternatively used to control osmolarity as it is also unmetabolizable by normal mammalian cells (Johnson et al., 1990; Ono et al., 2020). In contrast to the effects observed for EC, the total cell number of MSC cultured on TCP and GelMA-coated AUP2PEG scaffolds in hyperglycemic and hyperosmotic conditions (35.5 mM glucose) was not significantly reduced (Figure 11). Under normoglycemic hyperosmotic conditions (5.5 mM glucose +30 mM mannitol), MSCs cultured on GelMA-coated AUP2PEG scaffolds exhibited an increased proportion of PI-positive cells and noticeable morphological alterations. Cells cultured in TCP in the same medium, however, maintained their viability (more than 93% of viable cells) (Figure 11). Because Live/Dead staining cannot distinguish between osmotic stress, apoptosis, or membrane damage, these observations should be interpreted only as a reduction in overall viability. Nevertheless, hyperosmotic mannitol is known to induce endothelial and stromal cell stress responses, including apoptosis, nuclear shrinkage, and cytoskeletal rearrangements, according to previously published studies (e.g., Malek et al., 1998; Bálint et al., 2007). To more precisely determine the underlying mechanism, future work will incorporate targeted assays such as LDH release for membrane integrity and Annexin V/PI staining for apoptosis. The differences observed between 2D TCP and 3D GelMA-coated AUP2PEG scaffolds under hyperosmotic normoglycemic conditions are consistent with cellular osmotic stress responses rather than cell-type–specific effects. Hyperosmotic mannitol is known to induce EC shrinkage, nuclear condensation, cytoskeletal remodeling, and junctional alterations (Farkas et al., 2005; Bálint et al., 2007), which aligns with the reduced viability we observed in Live/Dead staining. In addition, although our study did not manipulate osmolarity to test hydrogel swelling directly, our measured swelling and gel fraction values reflect the highly hydrated and water-permeable nature of the AUP2PEG-GelMA constructs. In the broader hydrogel literature, environmental osmotic conditions such as ionic strength and solute concentrations are known to influence the hydration state and mesh characteristics of GelMA and other hydrophilic polymer networks (Vigata et al., 2021; De Piano et al., 2024). Thus, while we cannot attribute changes in viability to scaffolds’ osmotic responses without further testing, the combined observations suggest that cells in 3D hydrogels experience a different osmotic and biophysical environment than onto stiff 2D substrates, which may modulate their sensitivity to hyperosmotic mannitol. Because Live/Dead staining alone cannot separate osmotic, metabolic, or matrix-related contributions, these results should be interpreted only as evidence of reduced viability under hyperosmotic exposure.

## 4 Conclusion

In this study, we evaluated the cell-interactive properties of AUP2PEG scaffolds manufactured via digital light processing and subsequently coated with either GelMA or collagen-derived (atelocollagen) biomolecules using equine mesenchymal stromal cells and endothelial cells. XPS analysis demonstrated that physisorption of atelocollagen resulted in substantially lower protein deposition on the AUP2PEG surface compared to the uniform GelMA layer formed through photo-crosslinking. Importantly, these differences should not be interpreted as an intrinsic limitation of atelocollagen; rather, they reflect the limited efficiency of the physisorption strategy applied to surface modification of AUP2PEG-based scaffolds used herein. Because chemical crosslinking methods can disrupt collagen’s native triple helix or mask integrin-binding GXOGER motifs, potentially altering bioactivity, future work should focus on non-denaturing immobilization approaches capable of promoting robust collagen deposition while preserving the molecular domains essential for cell adhesion and signaling. Morphological analysis confirmed that GelMA formed a thin, continuous coating on the scaffold surface. This coating markedly improved the cell-interactive properties of AUP2PEG scaffolds: both EC and MSC exhibited substantially improved attachment and viability on GelMA-modified surfaces compared to non-coated controls. Moreover, the GelMA coating supported MSC trilineage differentiation, demonstrating that the functionalized AUP2PEG scaffolds can provide an appropriate microenvironment for both vascular and stromal cell functions. These findings collectively highlight the potential of GelMA-coated AUP2PEG scaffolds for constructing *in vitro* vascular wall models, while also identifying a clear pathway for improving collagen-based coatings through optimized, structure-preserving immobilization strategies. Furthermore, less senescence was observed when MSC were cultured on GelMA-coated AUP2PEG scaffolds. Further studies are required to elucidate the underlying mechanisms, e.g., by testing scaffolds with the same topology but different stiffness and evaluating the expression of pro-inflammatory cytokines and reactive oxygen species (ROS) production. To mimic hypeglycemic conditions, the GelMA-coated AUP2PEG scaffolds were incubated in a high glucose medium (35 mM glucose). Short-term hyperglycemic culture significantly reduced the number of viable cells attached to the surface for EC, but not for MSC. In addition, we observed detrimental effects of mannitol on cell viability, which is often used *in vitro* as an osmotic control when studying cell behaviour under high glucose conditions. This also should be further explored in future research. In conclusion, our results demonstrate that GelMA-coated DLP-printed AUP2PEG scaffolds support the growth of EC and MSC and can be used *in vitro* vascular wall models mimicking alterations induced by hyperglycemia.

## Data Availability

The raw data supporting the conclusions of this article will be made available by the authors, without undue reservation.

## Glossary

DLP: digital light processing
AUP2PEG: acrylate-endcapped urethane-based polymer precursor with a polyethylene glycol backbone
MSC: equine mesenchymal stromal cells
EC: equine endothelial cells
GelMA: gelatin methacryloyl
PEG: poly (ethylene glycol)
SEM: scanning electron microscopy
TCP: tissue grade culture plastic
PI: propidium iodide
SA: senescence-associated
HGM: media with high glucose concentration
MeAnH: methacrylic anhydride
ABAM: antibiotics-antimycotics solution
FBS: fetal bovine serum
EDTA: ethylenediaminetetraacetic acid
DPBS: Dulbecco’s phosphate-buffered saline (without calcium and magnesium)
DMEM-LG: low glucose DMEM
DAPI: 4′,6-diamidino-2-phenylindole
X-gal: 5-Bromo-4-chloro-3-indoxyl-β-D-galactosid
PBS: phosphate buffered saline
MWCO: molecular weight cut-off
Li-TPO-L: litium (2,4,6- trimethylbenzoyl) phenylphosphinate
^1^H NMR: proton nuclear magnetic resonance spectroscopy
TGA: thermo-gravimetric analysis
DSC: differential scanning calorimetry
UPW: ultrapure water
CAD: computer-aided design
XPS: X-ray photoelectron spectroscopy
Dil-Ac-LDL: 1,1′-dioctadecyl – 3,3,3′,3′-tetramethyl-indocarbocyanine perchlorate
PFA: paraformaldehyde
ANOVA: analysis of variance
PhI: photoinitiator
PA: photoabsorber
CAD-CAM: computer-aided design-computer-aided manufacturing
HUVECs: human umbilical vein endothelial cells
AGEs: advanced glycation end products
sGAGs: sulfated glycosaminoglycans.
