# Supplementary material for: Advancing in vitro vascular wall modelling using digital light processing to study hyperglycemia-driven cell changes
Source: Front Bioeng Biotechnol. 2026 Feb 4;14:1677364. doi: 10.3389/fbioe.2026.1677364 (PMC12913444; doi:10.3389/fbioe.2026.1677364)
Supplement: Supplementary file 1 [file DataSheet1.pdf]

Supplementary Material

1 Materials and Methods

Materials. Gelatin type B, isolated from bovine hides via an alkaline process, was supplied by Rousselot (Ghent, Belgium). Purified pepsin extracted collagen was provided by Dimitrios Zeugolis, Regenerative, Modular & Developmental Engineering Laboratory (REMODEL), University College Dublin (UCD), Dublin, Ireland. Methacrylic anhydride (MeAnH), polyethylene glycol (PEG) (2000 g mol^{-1}), isophorone diisocyanate, phenothiazine, triphenylphosphite, dimethyl terephthalate, antibiotics-antimycotics solution (100x) (ABAM) (A5955), collagenase type I (C9722), dispase II (D4693), dexamethasone (D2915), fetal bovine serum (FBS) (F7524), trypsin (T4799), ethylenediaminetetraacetic acid (EDTA) (E6758), trypan blue (T6146), L-ascorbic acid-2-phosphate (49752), β-glycerophosphate (G9422), Alizarine Red S (A5533); 3-isobutyl-1-methylxanthine (I7018), rh-insulin (I9278), indomethacin (I7378), rabbit serum (R4505), Oil Red O (O0625), low-gelling temperature agarose (A9414), Alcian blue (A3157); Nuclear Fast Red (N3020), resazurin sodium salt (R7017), Triton-X-100 (T8787) were obtained from Sigma-Aldrich (USA). Sodium hydroxide, potassium phosphate monobasic (KH_2PO_4), sodium phosphate dibasic (Na_2HPO_4), and glacial acetic acid were obtained from Chem-Lab NV (Zedelgem, Belgium). Butylhydroxytoluene was obtained from Innochem GMBH (Zossen, Germany). Neodecanoate was obtained from Shepherd Chemicals Co (Norwood, USA). Biosomer PEA-6 (MW 336 Da) was obtained from GEO Specialty Chemicals (Ambler, USA). Deuterium oxide (D_2O) and deuterated chloroform were obtained from Eurisotop (Saint-Aubin Cedex, France). Spectra/Por®4 dialysis membranes (Molecular weight cut-off (MWCO) of 12,000–14,000 Da) were obtained from Spectrum Chemical Mfg. Corp. (New Brunswick, USA). Speed Cure TPO-L (ethyl (2,4,6-trimethylbenzoyl) phenyl phosphinate) was purchased from Lambson (West Yorkshire, UK). Gentamycin (15710-049), Dulbecco's phosphate-buffered saline (without calcium and magnesium) (DPBS) (14190094), low glucose DMEM (DMEM-LG) (11880-036), were obtained from Gibco (USA). L-glutamine (25030-024) and calcein AM (65-0853-78) were supplied by Invitrogen (USA). EGM® -2 Endothelial Cell Growth Medium-2 Bullet Kit® (CC-3162), Human mesenchymal stem cell chondrogenic differentiation medium Bullet Kit ™ (PT3003), transforming growth factor-β3 (PT-4124) were purchased from Lonza (Switzerland). Acetylated low density lipoprotein labelled with 1,1′-dioctadecyl – 3,3,3′,3′-tetramethyl-indocarbocyanine perchlorate (Dil-Ac-LDL, L3484), SYTOX Blue (S11348), ActinGreen™ 488 ReadyProbes® Reagent (R37110), 4′,6-diamidino-2-phenylindole (DAPI) (202710500), D-(+)-glucose (410955000) were obtained from ThermoFisher Scientific (USA). MP Biomedicals (USA) supplied propidium iodide (PI) (25535-16-4). Mayer’s modified hematoxylin (ab220365) was obtained from Abcam (UK), and the calcium assay kit from Setinel Diagnostics (Italy). 5-Bromo-4-chloro-3-indoxyl-β-D-galactoside (X-Gal) (2315.1) was supplied by Carl Roth (Germany). D-mannitol (M0044) was supplied by TCI Europe NV (Belgium).

Material Synthesis

Synthesis of lithium (2,4,6-trimethylbenzoyl) phenylphosphinate (Li-TPO-L). The photoinitiator litium (2,4,6- trimethylbenzoyl) phenylphosphinate (Li-TPO-L) was prepared according to a protocol described earlier (Markovic et al., 2015). Briefly, 8.60 g (27.2 mmol) of (2,4,6-trimethylbenzoyl)-phenyl-phosphinic acid ethyl ester was dissolved in 150 mL butanone followed by the addition of 9.45

g (109 mmol) lithium bromide. The mixture was allowed to react for 24 h at 65°C under reflux conditions. The formed precipitate was isolated via suction filtration, washed with petroleum ether, and dried under vacuum at RT.

Material characterization

Thermal analysis. Thermal characterization of AUP2PEG was performed using thermo-gravimetric analysis (TGA) and differential scanning calorimetry (DSC). The TGA measurement was performed with a TA Instruments Q50 device (TA Instruments, USA) as described in (Pien et al., 2024). Briefly, around 10-25 mg of polymer was used for the measurement. After equilibration at 35 °C, the sample was heated to 750 °C at a rate of 10 °C·min^{-1} under an inert atmosphere (i.e. N_2). The results were analyzed using the TA Instruments Universal Analysis software.

DSC was performed to determine the melting and crystalization temperature of the synthesized AUP2PEG. The DSC measurement was performed with a TA Instruments Q2000 device (TA Instruments, USA) as described in (Pien et al., 2024). Briefly, samples (5-10 mg) for the DSC analysis were placed into T_{zero} aluminum DSC pans and subsequently sealed using an aluminum T_{zero} lid, equilibrated at 45°C prior to the start of the analysis, and heated at 10 °C·min^{-1} to 100°C. The results were analyzed using the TA Instruments Universal Analysis software.

2 Results and Discussion

Material synthesis and characterization. Diacrylate end-capped urethane-based PEG (AUP2PEG) was synthesized via a 2-step modification of PEG (2000 g·mol^{-1}) by an original protocol developed previously (Figure S1) (Houben et al., 2017). A ^{1}H-NMR spectrum of AUP2PEG and an overview of the physico-chemical properties of the synthesized AUP2PEG material are provided in Figure S2 and Table S1. Gelatin methacryloyl (GelMA) was obtained by modifying gelatin B with methacrylic anhydride (2.5 eq MeAnH with respect to the available primary amines) (Figure S3). According to ^{1}H-NMR spectroscopy (Figure S4), the degree of substitution of the developed GelMA was 99.7%.

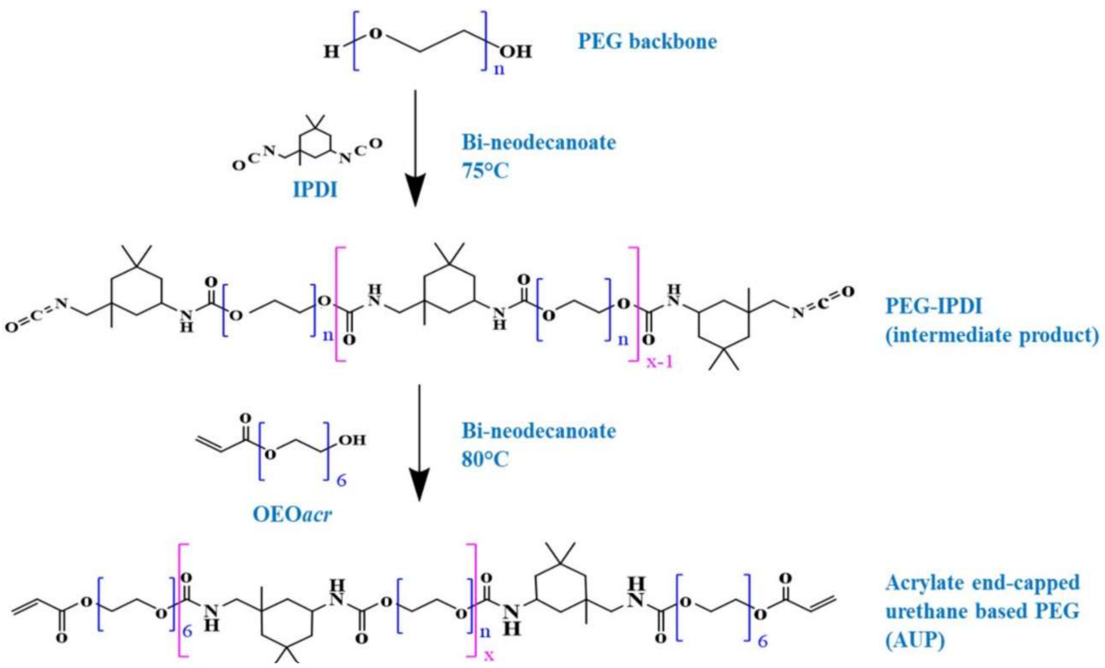

Figure S1. Reaction scheme for acrylate-endcapped urethane-based polymer (AUP) with a poly(ethylene glycol) (PEG) backbone (AUP2PEG) synthesis. Adapted from (Arslan, 2020), where n=45 for AUP2PEG (PEG2000) and x=3 on average. (PEG- poly(ethylene glycol), IPDI- isophorone diisocyanate, OEOacr- oligoethyleneoxide monoacrylate).

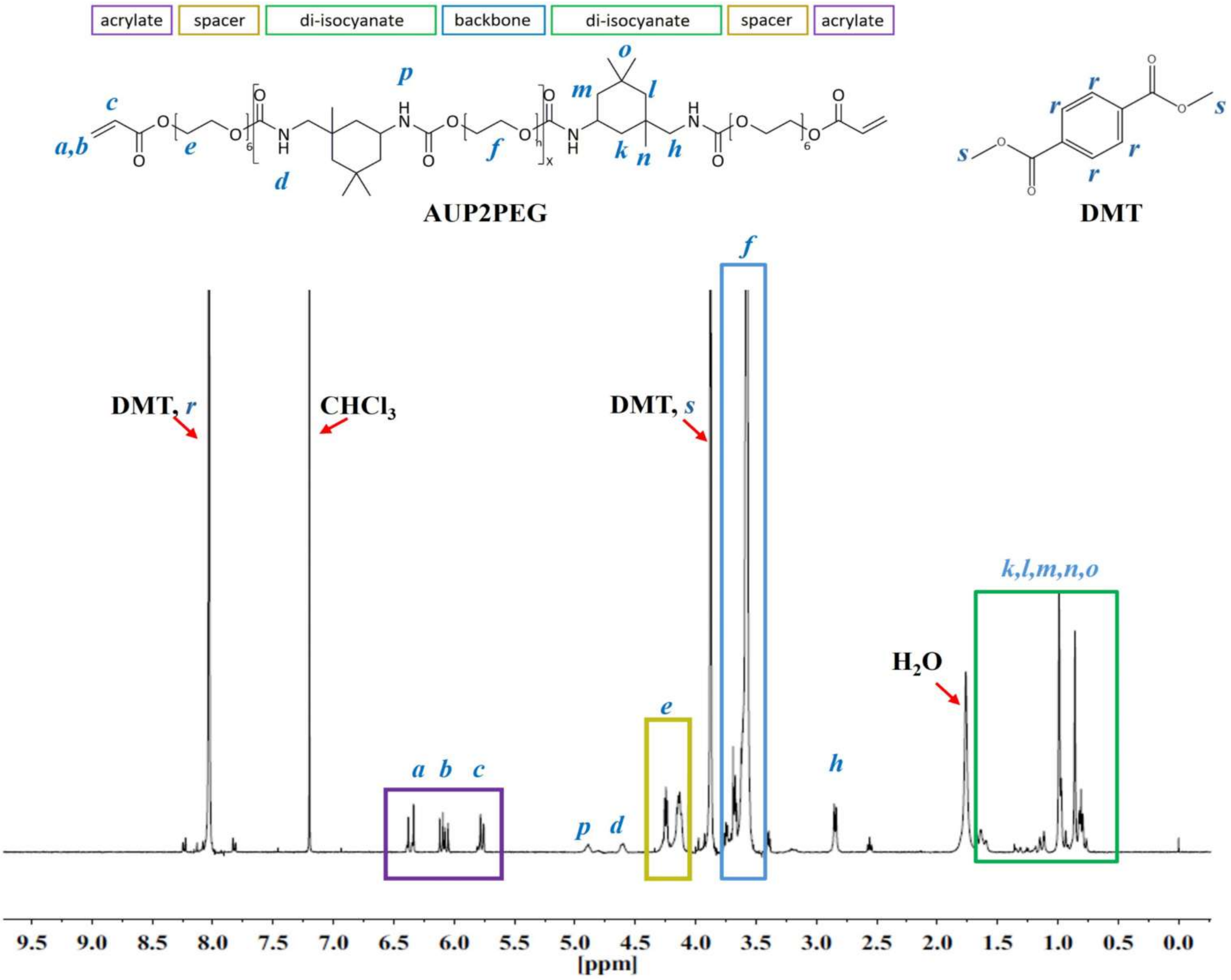

Figure S2. ^{1}H-NMR spectrum of AUP2PEG (dimethyl terephthalate (DMT) was used as an internal standard).

Table S1. Physico-chemical characterization of the synthetized AUP2PEG encompassing the acrylate content, molar mass (calculated from ^{1}H-NMR spectrum), the results of TGA, DSC analysis, gel fraction, swelling ratio, and Young's modulus

	C_{acr} (mmol g^{-1})	MM (g mol^{-1})	T_{95} (°C)	T_{onset} (°C)	T_m (°C)	T_c (°C)	GF (%), (for 30 wt%)	Swelling ratio ($g_{water}/g_{material}$), (for 30 wt%)	Young's modulus (MPa), (for 30 wt%)
**AUP2 PEG**	0.429	3974	295.3	364.2	33.6	6.37	99.95±0.03	3.6±0.038	0.738±0.03

C_{acr} - acrylate content; MM - molar mass; T_{95} - the temperature at 5% weight loss; T_{onset} - the onset degradation temperature; T_m - the melting temperature; T_c – crystallization temperature; GF- gel fraction.

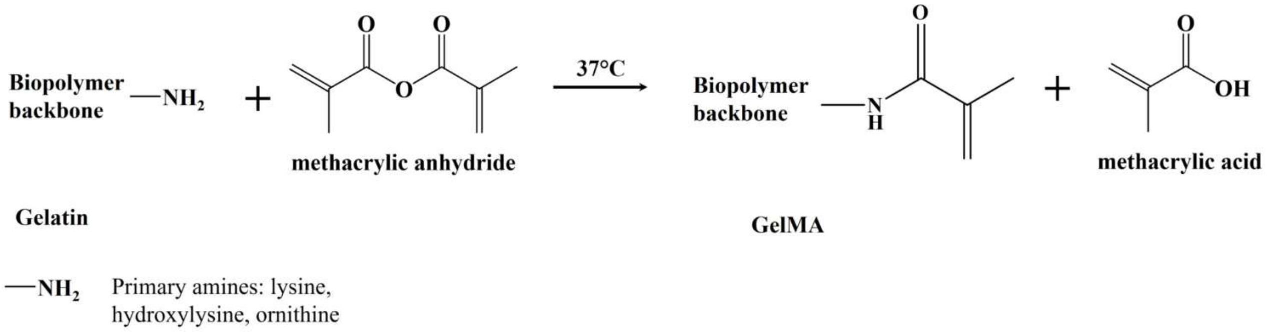

Figure S3. Development of gelatin methacryloyl (GelMA).

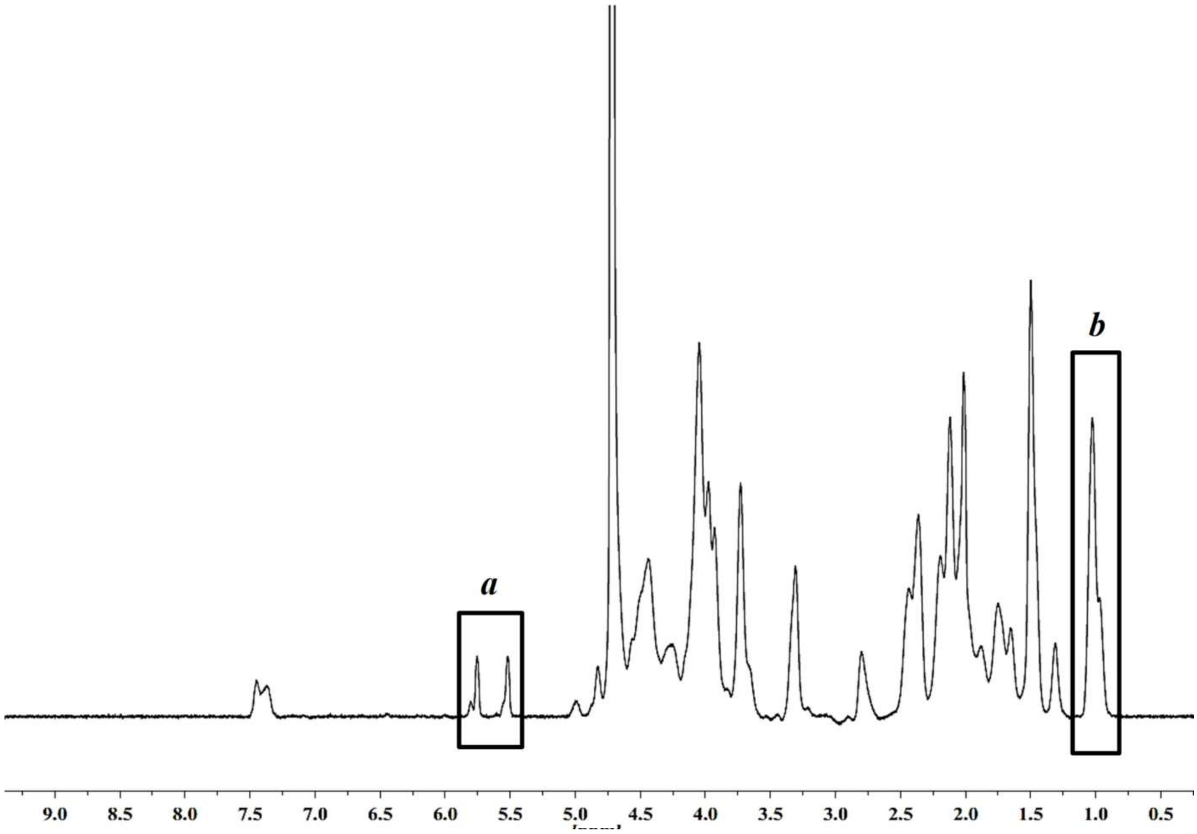

Figure S4. ^{1}H-NMR spectrum of GelMA. The peaks corresponding to the protons of the methacryloyl functionalities are indicated with ***a***. The peak that is marked with ***b*** corresponds to the hydrogens from the chemically inert valine, leucine, and isoleucine amino acids.

Optimization of AUP2PEG-based resin formulation for the DLP. The first step encompassed the evaluation of the processability of the resin compositions containing 30% (w/v) of AUP2PEG, and different concentrations of photoinitiator (Li-TPO-L, 5-10 mol% with respect to the acrylate content), and photoabsorber (tartrazine, 0.2-1 mol% with respect to the acrylate content). The disc CAD model that was used for the test of the processability of different resin compositions is presented in Figure 2A. An overview of the resin compositions tested and used printing conditions is given in supporting information Table S2. After equilibrium swelling in UPW, the diameter of manufactured disk scaffolds was measured using calipers, and disk thickness was measured from optical microscope images using FIJI (ImageJ) (n=6).

Table S2. Material processing and post-print processing. Resin compositions and printing parameters applied for DLP printing

Formulation composition							Printing parameters				
	**Group label**	**Acrylate content [mmol·g^{-1}]**	**w/v %**	**solvent**	**mol% PI***	**mol% PA***	**Laser power [%]**	**Irradiation time first layer [s]**	**Exposure time from working curve [s]**	**Exposure time for discs and dogbones [s]**	**Exposure time for tubular scaffolds [s]**
AUP2PEG	30/10/1				10	1		3	4.9	5	6
	30/10/0.5				10	0.5					
	30/10/0.2				10	0.2					
	30/05/1	0.429	30	H_2O	5	1	60	3	7.3	7.5	-----
	30/05/0.5				5	0.5					
	30/05/0.2				5	0.2					

*relative to the amount of acrylate endgroups in AUP2PEG . PI- photoinitiator; PA- photoabsorber.

The CAD/CAM mimicry ratio (%) at the X-Y plane (disc diameter) and the Z-plane (disc thickness) was calculated using the obtained data (Fig S5). Scaffolds developed using resin compositions containing 0.2 mol% of photoabsorber (for both concentrations of Li-TPO-L tested) and manufactured using the minimum required exposure time, exhibited polymerisation outside of irradiation zones (as visualised using optical microscopy). A possible reason for this is the diffusion of photoinitiated radicals, resulting in the lowest level of CAD/CAM mimicry of the fabricated scaffolds at the X-Y plane (Fig.S5).

The resin composition, containing 5 mol% of Li-TPO-L and 1 mol% of tartrazine, demonstrated the worst scaffold printing repeatability. The manufactured disc scaffolds exhibited a more pronounced variation between the samples in both the X-Y and Z planes.

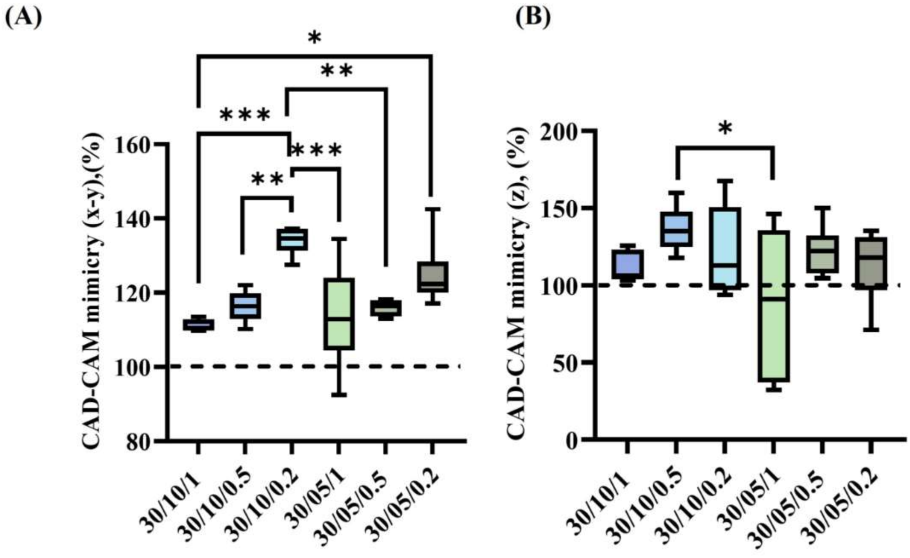

Figure S5. The repeatability of DLP-printed disc scaffolds using the different resin formulations. (**A**) CAD-CAM mimicry at the X-Y plane. (**B**) CAD-CAM mimicry at the Z-plane. All samples were measured after 72 hours of incubation in UPW at RT. Data were analyzed by one-way ANOVA with Tukey's post-hoc analysis. *p<0.05., ** p< 0.005, ****p<0.0001; (n=6).

For further comparison, we selected the two resin formulations (Table S3) that exhibited good CAD/CAM mimicry and printing repeatability in preliminary experiments, as described in the supporting materials.

Table S3. Optimized resin compositions and printing parameters applied for DLP printing

	**Formulation composition**						**Printing parameters**				
	**Group label**	**Acrylate content [mmol·g^{-1}]**	**w/v %**	**solvent**	**mol % PI***	**mol % PA***	**Laser power [%]**	**Irradiation time first layer [s]**	**Exposure time from working curve [s]**	**Exposure time for discs and dogbones [s]**	**Exposure time for tubular scaffolds [s]**
AUP2PEG	30/10/1	0.429	30	H_2O	10	1	60	3	4.9	5	6
	30/05/0.5				5	0.5		3	7.3	7.5	-----

*relative to the amount of acrylate endgroups in AUP2PEG. PI - photoinitiator; PA- photoabsorber

Aiming to select resin formulation/printing conditions that result in the scaffold exhibiting good CAD/CAM mimicry not only directly after printing, but also exhibiting the smallest changes of the scaffold geometry during further processing, we compared the CAD/CAD mimicry of the scaffolds directly after the printing process and after equilibrium swelling in UPW for 72 hours. The scaffolds were printed using the minimum required exposure time obtained from the working curves (Figure S6).

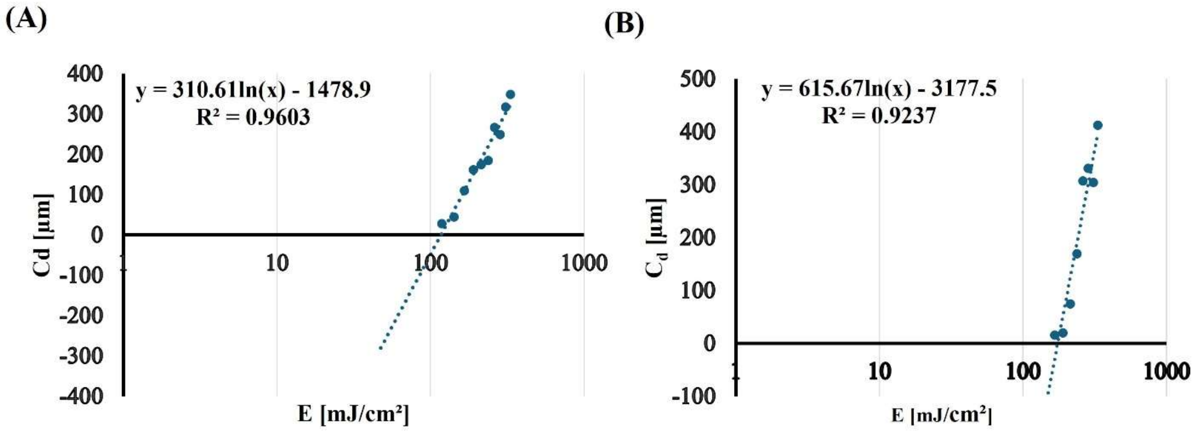

Figure S6. Working curves describing the relation between the applied dose (E) and the cured thickness (Cd) of the resins: (A) 30% (w/v) AUP2PEG, 10 mol% Li-TPO-L, 1 mol% tartrazine; (B) 30% (w/v) AUP2PEG, 5 mol% Li-TPO-L, 0.5 mol% tartrazine.

Our results indicate that scaffolds printed using a resin formulation containing 30% (w/v) AUP2PEG, 10 mol% Li-TPO-L, and 1 mol% of tartrazine (30/10/1) demonstrated less significant changes at X-Y plane after swelling in UPW as compared to the ones printed using a resin containing a lower concentration of PI and PA (30/05/0.5) (Figure S7 A,B).

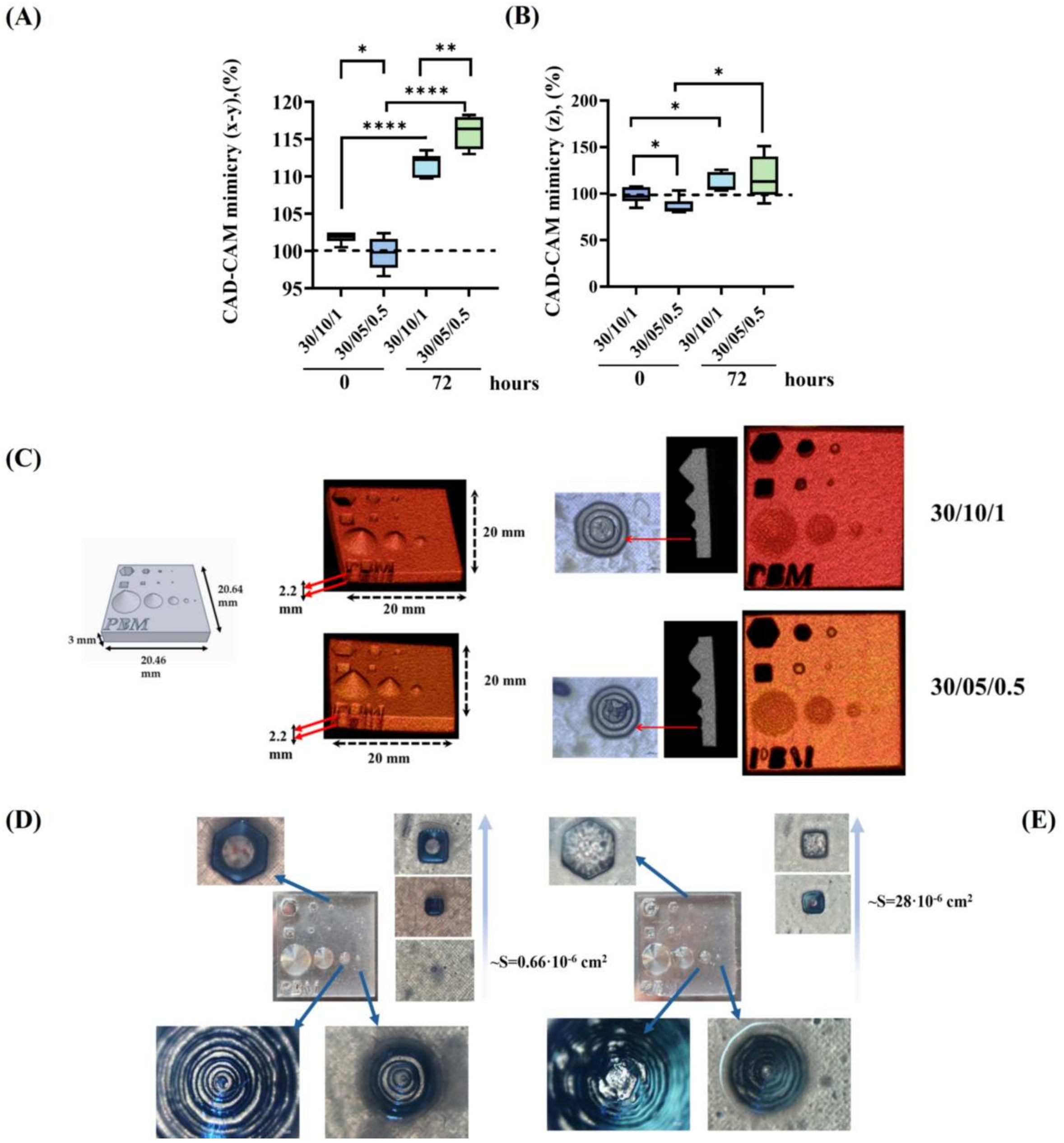

Figure S7. The CAD-CAM mimicry and the resolution of DLP-printed scaffolds using different resin formulations: CAD-CAM mimicry at the X-Y plane (**A**); at the Z-plane (**B**); The samples were measured directly after printing and after 72 hours of incubation in ultrapure water at RT; Printing parameters are presented in Table S3 (n=6). Data were analyzed by an unpaired *t*-test. *p<0.05, ** p< 0.005, ****p<0.0001; (**C**) The original CAD design of the benchmark, µCT images, and optical microscopy images of benchmarks directly after printing. (D) Optical microscopy images of the DLP-printed benchmark design with the different resin formulations, at the minimally required exposure time obtained from the working curve printed using 30/10/1; (E) printed using 30/05/0.5. Images are obtained from optical microscopy of the DLP-printed benchmark design in AUP2PEG in an equilibrium swollen state.

`

Next, we evaluated the resolution of the DLP prints by exploiting the different resin formulations using a benchmark designed to contain different structural elements, namely rectangular and hexagonal pores and cones (Figure S7 C, D ,E). The benchmarks were printed using the minimal exposure time calculated from the working curves for each resin formulation. The size and morphology of the printed scaffolds were evaluated in an equilibrium swollen state (i.e., after 72 hours of incubation in UPW, at RT). The results of the microscopic analysis of the scaffolds revealed that resin formulation 30/10/1 allowed the printing of smaller rectangular pores ($S_{30/10/1}$=$0.66 \cdot 10^{-6}$ cm^2 compared to $S_{30/05/0.5}$= $28 \cdot 10^{-6}$ cm^2) (Figure S7 D&E). Additionally, smaller hexagonal pores ($S_{30/10/1}$=0.145 cm^2) were obtained with the 30/10/1 formulation. However, in benchmarck printed using resin 30/05/0.5, rectangular pores of the same size were obstructed by the polymerised polymer. The printing accuracy of conical structures was also superior with the resin formulation 30/10/1.

The physico-chemical properties of the scaffolds were compared by measuring gel fraction (GF) and swelling capacity (SC). Scaffolds manufactured using each resin formulation demonstrated a high gel fraction (30/10/1- 89.26±0.89%, 30/05/0.5- 90±2.08 %), indicating efficient crosslinking of the polymer network. There were no significant differences detected in the swelling capacity of scaffolds from both groups (30/10/1- 3.8±0.05, and for 30/05/0.5- 3.9±0.1). Next, the mechanical properties of the scaffolds manufactured from both resin formulations were tested using tensile testing of the dogbone-shaped scaffolds (Figure 2B, Figure S8). Young's modulus, ultimate force, maximum stress, and total elongation were determined. There were no significant differences in mechanical properties between both resin formulations (Table S4).

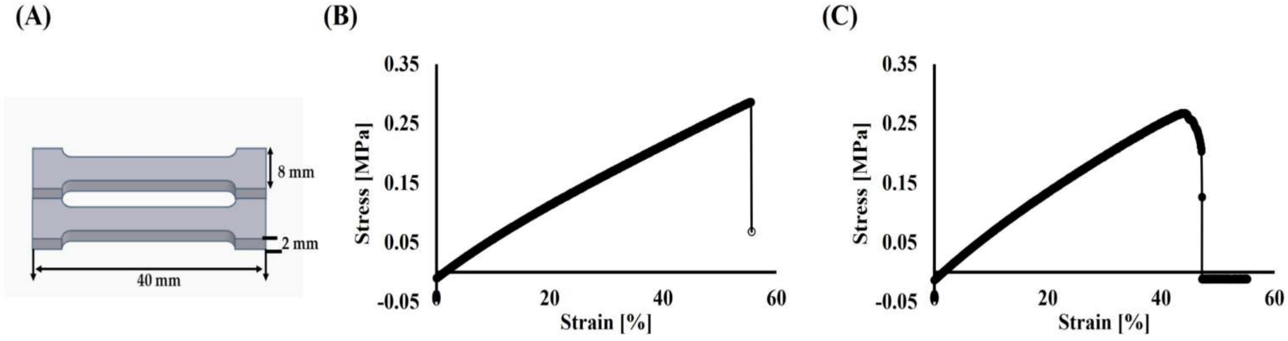

Figure S8. Example of a stress-strain curve obtained from uniaxial tensile testing of the DLP-printed dogbone-shaped samples: (**A**) CAD-model of dogbone-shaped samples for mechanical testing; (**B**) 30% AUP2PEG, 10 mol% Li-TPO-L, 1 mol% tartrazine; (**C**) 30% AUP2PEG, 5 mol% Li-TPO-L, 0.5 mol% tartrazine.

Table S4. Mechanical properties of the DLP-printed dogbone-shaped scaffolds

Resin formulation	Young's modulus (MPa)	Ultimate force (N)	Maximum stress (MPa)	Total elongation (%)
30/10/1	0.6±0.1	2.079±0.6	0.227±0.06	47.03±14.9
30/05/0.5	0.67±0.005	1.659±0.64	0.22±0.09	37.9±17.35

3 References

Arslan, A. (2020). Design and development of photo-crosslinkable urethane-based polymers for unprecedented scaffold manufacturing. Ghent: Universiteit Gent. Available at: https://biblio.ugent.be/publication/8684278 (Accessed October 19, 2025).

Houben, A., Roose, P., Van den Bergen, H., Declercq, H., Van Hoorick, J., Gruber, P., et al. (2017). Flexible oligomer spacers as the key to solid-state photopolymerization of hydrogel precursors. *Mater Today Chem* 4, 84–89. doi: 10.1016/j.mtchem.2017.01.005

Markovic, M., Van Hoorick, J., Hölzl, K., Tromayer, M., Gruber, P., Nürnberger, S., et al. (2015). Hybrid Tissue Engineering Scaffolds by Combination of Three-Dimensional Printing and Cell Photoencapsulation. *J Nanotechnol Eng Med* 6. doi: 10.1115/1.4031466

Pien, N., Deroose, N., Meeremans, M., Perneel, C., Popovici, C.-Ş., Dubruel, P., et al. (2024). Tailorable acrylate-endcapped urethane-based polymers for precision in digital light processing: Versatile solutions for biomedical applications. *Biomaterials Advances* 162, 213923. doi: 10.1016/j.bioadv.2024.213923
